# Robust thalamic nuclei segmentation using spectral clustering of fiber orientation distributions

**DOI:** 10.1371/journal.pone.0345649

**Published:** 2026-03-25

**Authors:** Debottama Das, Charles Iglehart, Ali Bilgin, Manojkumar Saranathan

**Affiliations:** 1 Department of Electrical and Computer Engineering, University of Arizona, Tucson, Arizona, United States of America; 2 Department of Radiology, University of Massachusetts Chan Medical School, Worcester, Massachusetts, United States of America; University of Calgary, CANADA

## Abstract

The thalamus comprises multiple nuclei that support higher-order cognitive functions. However, its internal architecture remains difficult to delineate using conventional T1- or T2-weighted MRI because of limited tissue contrast. Diffusion-weighted MRI provides richer microstructural detail, yet accurate segmentation is still challenged by low anisotropy and tissue heterogeneity. To address these challenges, we present a modified spectral clustering framework for thalamic segmentation. Our approach jointly leverages voxel-wise information and fiber orientation distribution (FOD) features derived from multi-shell multi-tissue constrained spherical deconvolution. When evaluated using spatial probabilistic maps that capture across-subject spatial variability in labels, k-means and spectral clustering exhibit broadly similar group-level variability patterns. However, the spectral clustering framework accommodates smaller thalamic subdivisions, including the lateral and medial geniculate nuclei (LGN and MGN), which required exclusion from the k-means configuration for stable parcellation. Under these conditions, spectral clustering achieved Dice scores of 0.73 for the mediodorsal–parafascicular (MD–Pf) complex and 0.51 for the ventral posterolateral (VPL) nucleus and produce a cluster corresponding to LGN. Furthermore, by combining structural and diffusion information, our approach enabled subdivision of the pulvinar into four distinct regions. These result position our modified spectral clustering as a robust and anatomically informed tool for thalamic clustering and pulvinar sub-segmentation.

## Introduction

The thalamus is a bilateral, subcortical gray matter structure that acts as a relay station between cortical and subcortical areas. It regulates various sensorimotor and cognitive functions, as well as sleep, consciousness, and alertness. The thalamus has a sophisticated architecture comprising multiple histologically and functionally distinct nuclei that are interconnected by intra-thalamic fibers [1]. Due to their distinct functionality, thalamic nuclei are critical to understanding many neurodegenerative and neurodevelopmental disorders.

Magnetic Resonance Imaging (MRI) is a non-invasive technique that provides enhanced visualization of soft biological tissues and enables automated segmentation and characterization of brain structures [2]. However, the intrinsic contrast provided by standard T1- and T2-weighted (T1w and T2w) MRI is insufficient for distinguishing thalamic nuclei, limiting its utility for automated segmentation. In contrast, Diffusion-Weighted MRI (DW-MRI) remains the only non-invasive imaging modality capable of depicting human brain white matter by describing the geometry of its underlying microstructure. DW-MRI captures the average diffusion of water molecules, thereby probing tissue structure at a scale much smaller than the imaging resolution and enables segmentation of each thalamic nucleus based on its unique fiber connections to the cortex. While state-of-the-art structural MRI–based tools such as THOMAS and FreeSurfer are widely adopted for automated thalamic parcellation, DW-MRI–based approaches provide a complementary avenue by leveraging microstructure or connectivity information. Consequently, automated thalamic parcellation methods based on DW-MRI fall into two groups: one that explores local diffusion properties [3,4] and another that examines global (long range) thalamic connectivity [5,6]. Fiber tracking approaches [5,6] based on global connectivity tend to generate segmentation patterns that differ from the Morel atlas [7].

Historically, diffusion tensor imaging (DTI)–based methods that rely on the principal eigenvector angle demonstrated that thalamic subdivisions can be delineated from local diffusion orientation [4]. However, because the thalamus is largely comprised of gray matter with relatively low fractional anisotropy, tensor angle-based metrics are unstable in regions with crossing or heterogeneous fiber orientations. This limitation motivated Battistella et al. [8] to introduce an orientation distribution function (ODF) [9]-based approach that segmented the thalamus into seven regions loosely aligned with its anatomical divisions. This method used a FreeSurfer-derived thalamus mask as the starting region of interest, with ODFs produced by Q-ball imaging [10]. Clustering was performed using a modified k-means algorithm on voxel coordinates and ODFs. Recently, Iglehart et al. [11] have demonstrated the feasibility of multi-shell multi-tissue constrained spherical deconvolution (MSMT-CSD) [12], which improves thalamic clustering performance by providing more reliable fiber orientation estimates in the thalamus.

While Battistella’s work highlighted the potential of ODF-based features for thalamic parcellation; however, three key limitations remain: (1) ODFs generated from Q-ball imaging are prone to noise and partial volume effects, limiting their reliability, (2) the reliance on FreeSurfer’s standard recon-all pipeline for whole thalamic mask generation is time-intensive and lacks critical nuclei like lateral geniculate nucleus(LGN) and medial geniculate nucleus (MGN), and (3) the use of modified k-means, which is highly sensitive to initialization and ill-suited for complex, non-spherical distributions. To overcome these limitations and building on the evidence provided by Iglehart et al., we propose a diffusion-based thalamic segmentation framework that integrates a fast segmentation method [13] for thalamus masking with MSMT-CSD to enhance the reliability of diffusion property estimation. Within this framework, we introduce a modified spectral clustering (SC) method. The proposed SC method is designed to more efficiently and effectively capture the complex, non-spherical organization of thalamic diffusion features, addressing shortcomings of k-means, while leveraging the strengths of MSMT-CSD.

Another outstanding challenge in thalamic nuclei segmentation is the subdivision of larger structures like the pulvinar nucleus. Given its extensive cortical connectivity and established role in higher-order visuospatial and cognitive functions, the medial pulvinar nucleus is emerging as key target for epilepsy deep brain stimulation (DBS) but poorly explored thus far. Although tractography and DTI studies have demonstrated the pulvinar nucleus’ widespread cortical and subcortical connectivity [14,15], evidence for topographically organized subdivisions in humans remains limited, with findings largely confined to visual and parietal territories [16]. To address this, we further applied the same FODs-based segmentation framework to the pulvinar nucleus, to delineate its internal subdivisions.

The proposed framework was evaluated using structural and diffusion MRI data from 30 healthy subjects drawn from the Human Connectome Project (HCP) Young Adult dataset. This dataset provides high-quality, multi-shell diffusion acquisitions in which images are collected at multiple b-values to sample diffusion signals across different diffusion sensitivities. This makes the dataset well suited for assessing advanced diffusion modeling and clustering strategies. The evaluation of the segmentation results was carried out both qualitatively and quantitatively.

## Materials and methods

### Data

Thirty healthy subjects selected at random from the Human Connectome Project (HCP) Young Adult cohort [17], aged between 22 and 36 years, were used for data analysis. This sample size was chosen to maintain computational feasibility for high-resolution structural and diffusion analyses. All imaging was performed on a customized Siemens Skyra 3T system equipped with high-performance gradients. Each subject has high-resolution structural T1w images with 0.7 mm isotropic voxels, providing detailed anatomical contrast. DW-MRI was acquired with 1.25 mm isotropic resolution across four shells (b = 0, 1000, 2000, 3000 s/mm²) and 90 diffusion directions, enabling robust fiber orientation reconstruction for connectivity analysis.

### Processing pipeline

**Fig 1:**
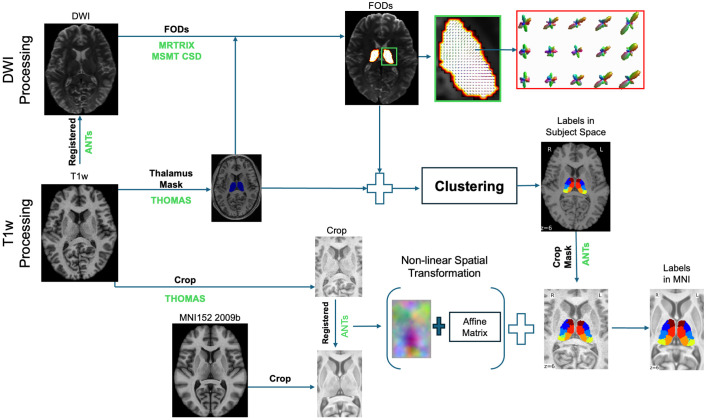
Thalamic Segmentation Workflow. Overview of the principal stages involved in thalamic segmentation using diffusion-weighted imaging (DWI) and T1-weighted (T1w) images, from preprocessing and feature extraction to clustering and label generation.

Fig 1 illustrates an end-to-end pipeline to parcellate the thalamus using combined T1w and DW-MRI. Brain-extracted T1w images were diffeomorphic registered to the b = 0 volume of the DWI using *antsRegistrationSyN.sh* (using -t s) [18], ensuring alignment between anatomical and diffusion spaces. The HIPS-THOMAS pipeline [13] was then applied to the registered T1w images to obtain whole-thalamic segmentations and corresponding cropped T1w volumes directly in diffusion space. Clustering was performed on the full registered volumes, and the resulting labels were then cropped to the T1w space defined by the HIPS-THOMAS output. For group-level analysis, the cropped T1w images were further registered to a cropped version of the ICBM 2009b T1w MNI template [19] using the same *antsRegistrationSyN.sh* workflow. The registration pipeline consisted of three stages: an initial rigid alignment, followed by affine registration, and a final nonlinear symmetric normalization step. The resulting transformations were subsequently applied to the cropped clustering-derived labels to generate MNI-space segmentations for group-level analysis.

Brain-extracted DWI images were reconstructed using MSMT-CSD [12] in MRtrix [20] to estimate white matter fiber orientation distributions (FODs). A maximum spherical harmonic (SH) order of 6 was used, resulting in 28 SH coefficients per voxel that characterize the angular profile of diffusion, consistent with Battistella et al. [8]. The thalamus mask, derived from the registered T1-weighted HIPS-THOMAS segmentation, was applied to extract these FODs from within the thalamus. The resulting voxel-wise FOD features along with voxel coordinates served as input for subsequent clustering.

### Clustering

**Fig 2:**
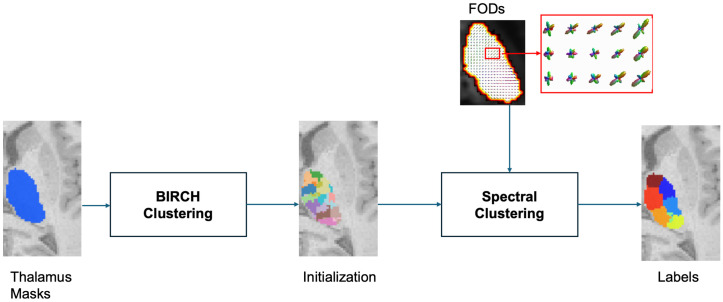
Workflow diagram illustrating clustering. The thalamus is first divided into small regions using BIRCH clustering for initialization, followed by spectral clustering to produce the final labels.

We introduce a modified spectral clustering [21] framework for thalamic parcellation, which forms the primary methodological contribution of this work. While the use of FOD-based features is adapted from prior works [11,22], we employ THOMAS-based thalamic masking as a fast alternative to FreeSurfer-based segmentation, substantially reducing preprocessing time. Our primary methodological contribution lies in incorporating a BIRCH-based initialization [23] into a standard spectral framework. Fig 2 illustrates the two-step procedure. In the first stage, BIRCH clustering is applied to the thalamic mask with a branching factor of 100 and a threshold of 1 to automatically generate superclusters that provide stable initialization. A threshold value of 1 was selected because values lower than 1 become computationally expensive, and this setting provides an effective balance that works well for both whole-thalamus and pulvinar-specific segmentation tasks.

The impact of this initialization on the final SC outcome is shown in S1 File. Direct voxel-level affinity matrix construction is computationally expensive ($O(n^{\text{2}})$ complexity for ~10,000 voxels per hemisphere) and highly sensitive to noise in low-anisotropy region. By grouping neighboring voxels into reproducible ~100–500 superclusters based on their spatial proximity, BIRCH reduces the problem size and provides stable initialization. This strategy mitigates sensitivity to initialization choices commonly encountered in centroid-based partitioning methods [24], while better preserving the non-spherical geometry of thalamic nuclei.

In the second stage, SC is performed on a nearest-neighbor affinity graph constructed from pairwise distances between superclusters, using the formulation of Battistella et al. [8], that combines spatial coordinates $c_{i}\in R^{\text{3}}$ and a MSMT-CSD–derived FODs as feature vectors $f_{i}\in R^{d}$. For any pair of voxels i and j, the total distance is defined as:


$$D_{tot}(i,j)=\alpha {∥c_{i}-c_{j}∥}_{\text{2}}+\;\gamma (\text{1}-\alpha ){∥f_{i}-f_{j}∥}_{\text{2}}\;$$ (1)

where α weights the contributions of the spatial coordinates and feature vectors, γ represents a scaling factor, and ${∥\;.∥}_{\text{2}\;}$denotes the Euclidean distance.

Given the disparity in value ranges between spatial coordinates and SH coefficients, γ was applied to the FODs to bring their Euclidean distances into a comparable range with the spatial distances. To select the gamma value, the absolute difference between the mean pairwise Euclidean distances of the spatial coordinates and feature vectors was minimized. This procedure yielded a final choice of γ=48 as shown in S2 in S1 File. In this work α is set to 0.5 based on Battistella et al. [8] and the boundary conditions are evaluated qualitatively as shown in S3 in S1 File.

The median inter-cluster distance between two superclusters $S_{p}$ and $S_{q}\;$is then given by


$$M_{pq}=median\left\{D_{tot}(i,j)|{i\in S}_{p},{j\in S}_{q}\right\}$$ (2)

The resulting matrix $M\in R^{m\times m}$encodes the distances between m superclusters.

For comparison, we also implemented the modified k-means algorithm originally proposed by Battistella [8],hereafter referred to as Battistella’s k-means, which utilizes a distance formula incorporating a combination of spatial coordinates and feature vectors as shown in Equation 1 and initialization using a data-driven approach consisting of 5,000 iterations to find the optimal starting centroids. The number of clusters was set at seven and nine for the Battistella’s k-means approach, with seven clusters chosen to be consistent with previous thalamic segmentation studies [5,6,8], excluding the LGN and MGN to ensure consistency with Battistella’s work [8]. Nine clusters were used for both Battistella’s k-means and SC, to account for the inclusion of the LGN and MGN within the thalamus mask.

For pulvinar segmentation, we employed the same processing pipeline for thalamic nuclei but with one modification: the full‐thalamus mask was replaced by the pulvinar mask also derived from HIPS-THOMAS. The γ parameter was determined using the distance-matching procedure as shown in S7 in S1 File, which yielded γ=35 for the pulvinar. The number of clusters was fixed at four based on the recent functional studies [25,26] and cytoarchitectonic Krauth-Morel atlas [7], enabling voxel grouping specifically within the pulvinar while preserving methodological consistency with the thalamic analysis.

### Evaluation

The absence of a definitive gold standard for thalamic nuclei complicates the evaluation of segmentation methods. For this study’s thalamus segmentation analysis, both quantitative and qualitative evaluations were performed.

For qualitative evaluation, we examined the maximum probability label maps and spatial probabilistic label maps in MNI space to visually assess cluster consistency, defined here as the degree to which labels overlap across subjects. The maximum probability label maps assign each voxel to the label most observed across subjects, providing a single group-level parcellation. In contrast, the spatial probabilistic label maps show the fraction of subjects assigned to each label at every voxel location. Together, the maximum probability label maps illustrate the dominant group pattern, while the spatial probabilistic label maps show how stable or variable the cluster boundaries are across subjects. Regions with sharp boundaries indicate high consistency, whereas fuzzier boundaries indicate greater spatial variation. Because the dataset is highly homogeneous (healthy control subjects 20–33 year old), these visual assessments provide a meaningful and reliable measure of inter-subject consistency rather than reflecting population-level anatomical variability.

Additionally, thresholded probability maps were generated by retaining only voxels with at least 50% label agreement across subjects, thereby emphasizing regions of high intersubject consensus. Clustering was determined to be consistent across subjects where labels remained spatially continuous at this threshold; conversely, the missing labels or fragmented boundaries served as visual indicators of high intersubject variability and inconsistency.

Quantitative analysis involved computing Dice similarity [27] scores between the generated clusters and two reference atlases: Krauth-Morel [7] and Allen Brain Human (ABH) [28] atlas. Details on the correspondence between atlas-defined thalamic nuclei and clustering-derived labels, along with a summary of atlas construction and label definitions, are provided in S1 Table. In addition to the Krauth–Morel and ABH atlases, we further evaluated the thalamic clustering results using the Saranathan atlas [29], with the corresponding confusion matrices shown in S5 Fig. To supplement the Dice scores, we also calculated the Adjusted Rand Index (ARI) [30] with Krauth-Morel atlas as reference. Unlike Dice scores, which requires an explicit one-to-one correspondence between labels, the ARI is uniquely suited for comparing partitions with different numbers of clusters. It provides a global measure of agreement by evaluating whether pairs of voxels are consistently grouped together across both the clustering solution and the anatomical reference, regardless of the total number of labels. In practice, ARI values range from −1–1, where values close to 1 indicate strong agreement between voxel groupings, values near 0 indicate chance-level agreement, and negative values indicate agreement worse than chance.

For pulvinar clustering, qualitative evaluation was again employed through visual inspection of the maximum probability and spatial probabilistic label maps in MNI space. Recent studies show different spatial organization that is different from Krauth-Morel’s subdivisions [25,26,31]; therefore, rather than relying solely on Dice scores comparisons with the Krauth-Morel atlas, we evaluated spatial overlap using Dice scores against both the Krauth-Morel and ABH Atlas, and further characterized structural connectivity patterns to support the biological plausibility of the resulting subdivisions. We employed a tractography based analysis to map the structural connections of each pulvinar clusters with cortical [32] targets, thereby characterizing each cluster’s connectivity patterns.

This analysis was performed on a subset of five subjects (shown in S6 Fig.) chosen at random, as high resolution whole brain tractography is computationally expensive. For each subject, 100 million streamlines were generated using MRtrix [20] (tckgen) to ensure sufficient sampling of long-range thalamo-cortical pathways and stable estimation of connection patterns. Streamlines were then summarized using tck2connectome (scaled using inverse volume weighting) on a subject-by-subject basis. Connectivity was quantified for pulvinar subdivision from Krauth-Morel atlas transformed into subject space, and pulvinar parcellation from modified SC. This setup enabled consistent comparison of pulvinar–cortical connectivity patterns across subjects while maintaining computational feasibility.

Based on prior studies [25,26,31,33], we focused on the major cortical connectivity patterns of the pulvinar. The anterior pulvinar is primarily connected with prefrontal and cingulate cortices, supporting executive and attentional control. The medial pulvinar shows strong associations with temporal association areas and limbic regions, consistent with its role in higher-order integrative and emotional processing. The inferior–lateral pulvinar is closely linked with parietal association areas, the dorsal visual stream, and occipital cortices, reflecting its involvement in visuospatial and visual recognition pathways.

### Statistical analysis

Quantitative evaluation was primarily based on the Dice similarity score, which was used to assess spatial correspondence between the clustering results and atlas-defined thalamic nuclei. Dice-based analyses were performed for Battistella’s k-means clustering with nc = 7 and nc = 9 (evaluated against the Krauth–Morel atlas), as well as for Battistella’s k-means (nc = 7) versus SC (nc = 9), evaluated against both the Krauth–Morel and ABH atlases.

To enable comparison across methods with differing numbers of clusters, each cluster was matched to atlas nuclei using a maximum Dice criterion. Nucleus-wise comparisons were then performed using Wilcoxon signed-rank tests across subjects to identify subdivisions exhibiting statistically significant differences between clustering methods, with significance assessed at α=0.05.

In addition, Adjusted Rand Index (ARI) was used as a complementary measure of inter-subject agreement for comparisons of Battistella’s k-means cluster cardinality, as ARI is insensitive to label permutations and robust to differences in cluster cardinality. Post-hoc pairwise comparisons of ARI values were conducted using Wilcoxon signed rank test assessed at α=0.05.

We further studied the inter subject consistency of the four pulvinar subdivision produced by SC across the 30 subjects (S8b in S1 File). For each subject, we calculated: (1) the volume ratio of each cluster relative to the total pulvinar volume and (2) the centroid distance to the pulvinar boundary, obtained via a Euclidean distance transform and converted to millimeters using the image affine. (3) Finally, inter subject variability is summarized using coefficients of variation.

## Results

Our results are organized into two parts: the first part focuses on the performance of the modified SC on whole thalamus subdivision (i.e., thalamic nuclei), and the second part evaluates the pulvinar subdivision.

### Thalamic parcellation

#### Sensitivity analysis of k-means cluster cardinality.

To examine the effect of cluster cardinality on Battistella’s k-means clustering, we compared solutions with nc = 7 and nc = 9. As shown in S4a in S1 File, the group-level probability maps, displayed at a 50% probability threshold, provide a qualitative visualization of voxel-wise cluster agreement across subjects. Regions that do not consistently receive the same cluster label across subjects are suppressed at this threshold, highlighting areas of higher inter-subject consistency. Given the homogeneous nature of the dataset, limited inter-subject variability is expected; However, compared to nc = 7, the nc = 9 solution produces less consistent regions, with cluster labels varying more across subjects as the number of clusters increases, resulting in a larger fraction of voxels with missing label denoted by red arrows in S4a in S1 File.

Quantitative evaluation using Dice scores (S4b in S1 File) revealed largely comparable performance between the two configurations across most thalamic nuclei. The nc = 7 solution showed higher Dice scores for AV and pulvinar regions, whereas nc = 9 achieved higher Dice scores for VLa. To further assess inter-subject consistency beyond region-wise overlap, clustering stability was evaluated using ARI. The k-means nc = 7 configuration achieved a mean ARI of 0.356 ± 0.039, whereas increasing the number of clusters to nc = 9 resulted in a lower mean ARI of 0.317 ± 0.031. This reduction in ARI was statistically significant (p < 0.001), indicating a degradation in inter-subject agreement at higher cluster resolution despite comparable Dice scores in selected nuclei. Based on this analysis and to be fair to Battistella’s original work, we decided to use nc = 7 configuration for the k-means analysis in all subsequent experiments.

#### Qualitative evaluation.

**Fig 3:**
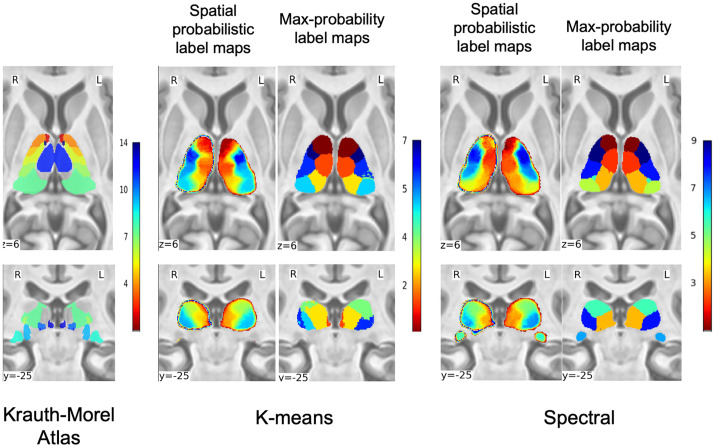
Qualitative Analysis of Thalamic Clustering Algorithms. Axial (z = 6 mm) and coronal (y = –25 mm) sections on the ICBM 2009b T1-weighted template comparing the Krauth-Morel atlas and thalamic parcellation strategy. For each method, the first column shows the spatial probabilistic label maps, and the second column shows the corresponding maximum-probability label maps.

Fig 3 presents the corresponding maximum probability label maps and spatial probabilistic label maps in MNI space. Both methods show visually consistent spatial patterns with low intersubject variability for SC at nc = 9 and Battistella’s k-means at nc = 7 across subjects. LGN was clearly identified by SC and showed consistent representation across subjects. The LGN and MGN had to be removed from the thalamus mask for k-means at nc = 7 for consistent clusters across the subjects. The role of initialization was further examined in S1 in S1 File, which showed that SC with BIRCH initialization yielded more spatially stable subdivisions than SC without initialization.

#### Quantitative evaluation.

**Fig 4:**
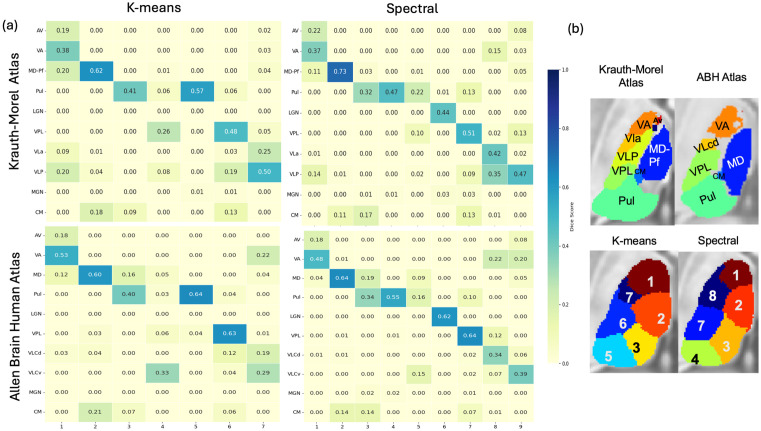
Evaluation of thalamic segmentation accuracy. **(a)** Confusion matrices showing Dice scores between the maximum-probability label maps generated by each clustering method and the reference atlases (Krauth–Morel and Allen Brain Human Atlases). **(b)** Overlay of maximum-probability label maps and atlas labels on an MNI template, illustrating the anatomical correspondence between data-driven clusters and atlas-defined thalamic nuclei.

To quantify anatomical correspondence, we computed Dice scores between each method’s maximum probability label map and two anatomical reference atlases: the Krauth–Morel atlas and the ABH atlas. Fig 4a. compares the confusion matrices produced by each clustering method. To identify the correspondence between clusters and anatomical nuclei, Fig 4b shows the right thalamus maximum-probability label maps overlaid on the MNI template alongside the Krauth-Morel atlas, with cluster indices indicated on the data-driven maps and corresponding thalamic nucleus labels shown on the atlas, illustrating the anatomical correspondence between the clustered solutions and atlas-defined nuclei.

Against the Krauth–Morel atlas, k-means peak overlaps of 0.62 for the MD-Pf complex, 0.57 for the pulvinar, 0.48 for VPL, and 0.50 for VLP, with lower values for VLa (0.25). In comparison, the SC produced a higher maximum overlap for MD-Pf (0.73) and VLa (0.42), and a comparable value for VPL (0.51). Pulvinar overlap in the SC was distributed across multiple clusters, with values of 0.32, 0.47, and 0.22, rather than concentrated in a single cluster. The LGN showed a non-zero overlap of 0.44 under SC.

When evaluated against the ABH atlas, k-means achieved overlaps of 0.60 for MD, 0.64 for the pulvinar, and 0.63 for VPL. SC yielded overlaps of 0.64 for MD, 0.55 for the pulvinar, and 0.64 for VPL, with pulvinar overlap distributed across multiple clusters (0.34, 0.55, and 0.16). In this atlas, the LGN exhibited a clear overlap of 0.62 in the SC. Additional VL-related subdivisions (e.g., VLCd and VLCv) showed moderate overlap values ranging from 0.34 to 0.39 in SC.

The evaluation against the Saranathan atlas reveals overlap patterns that are consistent with those observed for the Krauth–Morel atlas. In particular, the relative correspondence of major nuclei such as MD–Pf, VPL, and VLP is preserved across methods, while the pulvinar is subdivided into three components in the SC, with overlap distributed across multiple clusters. Although absolute overlap values vary due to differences in atlas definitions and labeling conventions, the overall behavior of both clustering methods remains stable across reference atlases.

Table 1 summarizes the maximum Dice scores obtained for each thalamic nucleus by matching each atlas label to its best corresponding cluster, evaluated separately using the Krauth–Morel atlas and the Allen Brain Human Atlas. Statistical significance was assessed using a Wilcoxon signed-rank test across subjects. Using the Krauth–Morel atlas, SC showed statistically significant improvements for MD–Pf and VLa, while k-means exhibited significantly higher Dice scores for the pulvinar. No statistically significant differences were observed between the two methods for AV, VA, VPL, VLP, or CM. Non-zero overlap for LGN and MGN was observed only in the SC configuration.

**Table 1:** Per-nucleus statistical comparison of Dice scores between k-means (nc = 7) and spectral clustering (nc = 9) using Wilcoxon signed ranked test.

Atlas	Nucleus	K-means Dice	Spectral Dice
**Krauth-Morel Atlas**	AV	0.212	0.226
	VA	0.392	0.392
	MD-Pf	0.600	0.691***
	Pul	0.573***	0.489
	LGN	–	0.475***
	VPL	0.522	0.499
	VLa	0.266	0.355***
	VLP	0.510	0.472
	MGN	–	0.08***
	CM	0.267	0.280
**Allen Brain Human Atlas**	AV	0.228	0.249
	VA	0.621***	0.541
	MD	0.615	0.680***
	Pul	0.660***	0.574
	LGN	–	0.711***
	VPL	0.686*	0.651
	VLcd	0.261	0.378***
	VLcv	0.579 *	0.496
	MGN	–	0.068***
	CM	0.282	0.267

*p < 0.05, *** p < 0.001; Wilcoxon signed-rank test.

A similar pattern was observed when using the ABH atlas. SC showed statistically significant differences for MD and VLcd and again yielded non-zero overlap for LGN and MGN. In contrast, k-means showed statistically significant increase for VA, pulvinar, VPL, and VLCv. No significant differences were observed for AV or CM.

Across both reference atlases, absolute Dice scores varied with atlas definition; however, the overall pattern of nuclei showing significant differences was consistent.

### Pulvinar subdivision

#### Qualitative evaluation.

**Fig 5:**
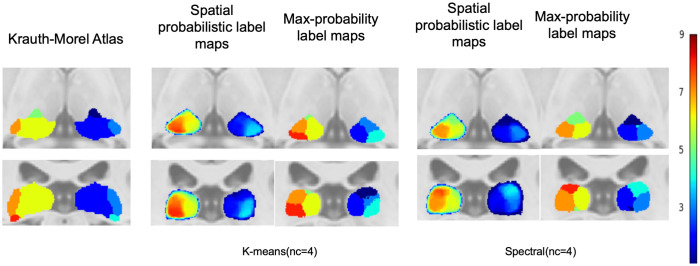
Qualitative Analysis of Pulvinar Parcellation. Spatial probabilistic label maps and corresponding maximum-probability label maps projected onto the ICBM 2009b T1-weighted MNI template at the axial slice (z = 6 mm) and coronal slice (y = −29). Results are shown for the Krauth–Morel atlas (left), k-means clustering (center), and spectral clustering (right), illustrating qualitative differences in pulvinar parcellation.

In this part, we evaluate pulvinar subdivision performance. Fig 5 shows maximum‐probability and spatial‐probability label maps to convey segmentation confidence and spatial uncertainty across the cohort using Battistella’s k-means and Spectra clustering. At the subject level, the segmentation produced reproducible subdivisions across individuals, with boundaries remaining stable for SC showing more stable boundaries, reflected by reduced boundary fuzziness in the spatial probabilistic label maps. In contrast, k-means showed increased boundary fuzziness, indicating greater intersubject variability. The role of initialization in pulvinar parcellation was further examined in S8a Fig. SC with BIRCH initialization successfully divided the pulvinar into four anatomically meaningful subdivisions, whereas only three clusters were obtained without initialization.

#### Quantitative evaluation.

**Fig 6:**
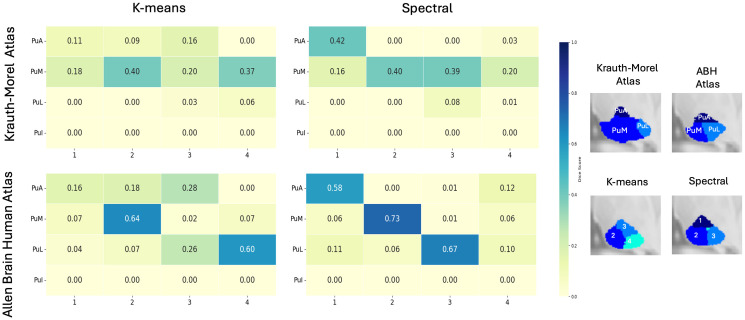
Pulvinar Dice Score Analysis Across Reference Atlases. Dice score confusion matrices between clustering-derived pulvinar subdivisions and the reference atlases, Krauth–Morel (top) and Allen Human Brain Atlas (bottom). Results are shown for k-means (left) and spectral clustering (right). Rows correspond to atlas labels, and columns correspond to cluster labels.

Fig 6 shows Dice score matrices comparing clustering-derived pulvinar labels with two anatomical atlases: Krauth–Morel atlas (top row) and ABH atlas (bottom row), with k-means results on the left and SC on the right. Rows represent atlas-defined pulvinar subdivisions and columns represent clustering-derived labels. Across both atlases, SC demonstrates significantly higher Dice scores than k-means. This is most evident for ABH atlas, where SC yields higher peak Dice scores with clearer subdivision-to-cluster correspondence, whereas k-means shows more distributed and mixed overlaps. Based on the Dice scores with the ABH atlas, Cluster 1 maps to PuA, 2 maps to PuM,3 maps to PuL. The fourth cluster shows certain overlap with all three regions. Overall, the results indicate that SC outperforms k-means in recovering atlas-consistent pulvinar subdivisions.

To assess anatomical-functional relationships, we used MRtrix’s tck2connectome to derive structural connectivity profiles between each cluster and cortical and subcortical regions in five subjects. We refer to the right hemisphere clusters as R1–R4 and the left hemisphere clusters as L1–L4. Analysis of the connectivity matrix derived from the SC–based pulvinar segmentation reveals quantitatively distinct connectivity profiles across clusters as shown in S9 Fig. In the left hemisphere, L1 shows elevated connectivity with sensory and motor cortices, with values of 4.93 for left sensory and 2.08 for left motor regions, while connectivity to the amygdala–hippocampus (AH) remains low (0.29). L2 exhibits the highest connectivity with AH, reaching 4.88, with additional but lower-magnitude connections to visual (1.51) and frontal–parietal regions (~1.3). L3 shows modest connectivity to visual and amygdala-hippocampal regions, with values of ~0.96 for visual cortex and 1.87 for AH, while remaining below 2.0 across other cortical and subcortical targets. L4 shows intermediate connectivity, with elevated values to left sensory (3.88) and AH (3.59) regions.

A similar pattern is observed in the right hemisphere. R2 demonstrates the highest AH connectivity (4.87), whereas R4 shows higher connectivity to sensorimotor regions, including right sensory (3.43) and right motor (1.94). R3 again exhibits comparatively lower and more distributed connectivity, with all target values below ~2.1. These findings suggest that, beyond atlas overlap, the SC–based parcellation yields internally distinct connectivity profiles, offering an additional perspective on the relevance of the derived subdivisions.

The SC clusters differ substantially in volume and spatial extent from atlas-defined pulvinar nuclei, resulting in systematically different connectivity strengths across parcellation schemes. Consequently, connectivity varies between the SC-based and atlas-based connectomes, reflecting the effects of inverse node volume normalization rather than reduced biological connectivity. Importantly, the relative connectivity patterns across SC clusters remain consistent and interpretable.

## Discussion

In this study, we introduced a modified spectral clustering framework for thalamic parcellation and demonstrated its advantages over the modified k-means clustering proposed by Batistella et al. [8]. By integrating HIPS-THOMAS for efficient thalamic masking with MSMT-CSD for feature extraction, we developed a preprocessing pipeline that was more than fourteen times faster than Battistella’s pipeline, enabling scalability to large neuroimaging datasets. This acceleration is primarily attributable to the avoidance of FreeSurfer-based processing and the elimination of the large number of k-means iterations (≈5000) previously required to achieve clustering stability. Across multiple evaluations, SC consistently produced parcellations that were generally comparable to k-means, with more consistent spatial organization and anatomical agreement in selected regions. The method achieved higher Dice scores for well-defined nuclei such as VPL and MD-Pf and partially recovered smaller nuclei, including LGN, which were excluded in Battistella’s k-means approach. Furthermore, the pulvinar nucleus was reproducibly subdivided into four clusters across subjects, with connectivity profiles that aligned with those from classical histological atlases.

Two design choices were central to these gains over Battistella’s k-means [8]. The use of MSMT-CSD enhanced orientation reliability in regions with complex tissue composition, contributing to improved clustering stability. Iglehart et al. [11] earlier reported that MSMT-CSD outperforms q-boot reconstruction for thalamus segmentation, achieving higher anatomical fidelity and reduced noise. Second, we employed a two-stage clustering in which BIRCH generates spatially coherent superclusters, followed by SC on a supercluster affinity graph. This design lowers the computational burden and minimizes sensitivity to random initialization. The supercluster abstraction helps to smooth voxel scale noise in regions of low anisotropy within the thalamus while also preserving the non-spherical geometry of nuclear boundaries that are usually difficult for centroid based methods to capture.

Our analyses showed that SC produced improvements over modified k-means. SC delivered higher Dice scores for nuclei such as MD–Pf and VLa, and it was able to partially recover the LGN, which the seven-cluster k-means configuration necessarily excluded. Attempts to incorporate LGN and MGN within the k-means framework led to unstable parcellations, with inconsistent clusters across subjects. While modified k-means demonstrated comparable performance for VPL and VLP and a statistically significant advantage for the pulvinar (p < 0.001), for both Battistella’s k-means and spectral clustering the pulvinar was subdivided into multiple clusters (two for k-means and three for SC). Consequently, assessing pulvinar performance solely based on the maximum Dice score does not fully capture the underlying parcellation behavior and may overemphasize alignment with larger nuclei where centroid-based partitioning benefits from more distinct diffusion contrast. For smaller or less well differentiated nuclei such as AV,VA, and CM, both approaches remained challenged, consistent with known limitations of diffusion MRI such as limited spatial resolution and low anisotropy in thalamic gray matter [4,34]. Overall, SC provided modest but consistent improvements across subjects, with reduced intersubject variance even after considering LGN and MGN nuclei.

Applying the same framework locally to the pulvinar yielded four reproducible subdivisions per hemisphere, while k-means failed to produce anatomically meaningful subdivision. Group-level probability label maps showed crisp territorial separation, and tractography-based profiles indicated differentiated coupling with visual, sensorimotor, and limbic-temporal systems. Consistent with prior functional connectivity-based parcellations of the human pulvinar [25,33], SC derived subdivisions do not exhibit one-to-one correspondence with Krauth–Morel nuclei. Both resting-state functional connectivity–based [33] and hybrid structural–functional [25] studies have shown that the anterior pulvinar is relatively homogeneous and maps coherently to a single cluster, whereas the medial pulvinar is functionally heterogeneous and distributed across multiple clusters. In contrast, lateral and inferior pulvinar territories often show partial convergence, reflecting shared visual connectivity profiles rather than distinct anatomical boundaries. Within this framework, the assignment of L1/R1 to PuA, L2/L4 and R2/R4 to PuM, and L3/R3 to a combined PuL–PuI region is consistent with previously reported connectivity-driven organization of the pulvinar.

This study had several limitations. First, diffusion MRI acquired even at 1.25 mm resolution with standard b values imposes a fundamental constraint on resolving very small thalamic nuclei and fine intra-thalamic laminae and this will be worse at typical 2 mm spatial resolution used in standard neuroimaging protocols. In addition, partial volume mixing at the thalamic boundaries, where gray matter and cerebrospinal fluid are often present alongside penetrating white matter fibers, can bias the estimated FOD profiles and reduce the reliability of clustering. Such effects are particularly pronounced for small or low-contrast nuclei such as AV and CM, which likely explain their lower Dice scores. Second, our evaluation relied on correspondence with atlases rather than subject-specific histological ground truth. This reliance on atlases may not fully account for inter-individual anatomical differences. The reported Dice should therefore be interpreted as reflecting anatomical plausibility rather than absolute accuracy. In addition, differences in parcellation strategies and nomenclature across thalamic atlases introduce further sources of variability when interpreting atlas-based comparisons, as demonstrated by previous works [35,36]. Third, the weighting between spatial coordinates and FOD features was tuned empirically and demonstrated robust performance in our dataset, but this balance may vary with different scanners, acquisition protocols, or clinical populations. Fourth, our comparative evaluation focused on Battistella’s k-means implementation, which employs data-driven centroid initialization. While this represents a widely used baseline, recent study [37] has also explored atlas-informed centroid initialization strategies that may yield different clustering behaviors and warrant future investigation. Fifth, tractography-based validation is inherently limited by its sensitivity to dominant fiber bundles and its reduced ability to capture smaller or crossing pathways, which may lead to underrepresentation of subtle pulvinar connections. Finally, our study was conducted in healthy young adults; a relatively homogeneous population in which inter-individual anatomical variability is expected to be minimal compared to aging or disease cohorts. However, clinical populations are likely to exhibit greater anatomical heterogeneity, making it important to distinguish between clustering inconsistency arising from methodological limitations versus genuine individual anatomical differences in thalamic organization. Further validation across aging and disease cohorts is necessary to establish the generalizability and clinical applicability, and subject-specific clinical outcomes could help differentiate these sources of variability.

The findings of this study indicate that the modified spectral clustering can serve as an automated post-processing step appended to THOMAS pipeline to provide a ready-to-use solution for generating four anatomically consistent pulvinar subdivisions in addition to whole thalamus labels. From a methodological perspective, several directions hold promise for further refinement. First, the development of adaptive, subject-specific strategies for balancing spatial coordinates and FOD features could help reduce sensitivity to parameter selection. Second, more advanced graph construction techniques that employ self-tuned kernels or data-driven affinity learning may further enhance the stability of the clustering. Third, the integration of complementary modalities, such as susceptibility-weighted imaging or quantitative T1 and T2 maps, could provide additional priors to improve the delineation of smaller nuclei. From an application standpoint, the availability of reliable pulvinar sublabels offers an important opportunity to advance studies of thalamo-cortical dysconnectivity in movement disorders, visual attention, and limbic functions, while also supporting individualized targeting in clinical contexts such as deep brain stimulation planning, stereotactic radiosurgery, and intraoperative navigation, where long-range tractography may be limited by time constraints, lower-quality clinical acquisitions, or disruption of white matter pathways by lesions.

In summary, a systematic combination of MSMT-CSD feature modeling, supercluster-based graph reduction, and spectral clustering yields thalamic and pulvinar segmentations that are reproducible, anatomically interpretable, and operationally scalable. The proposed framework outperforms the modified k-means approach of Battistella et al. in terms of robustness and anatomical plausibility, while offering a scalable pathway for broader research and potential clinical applications.

## Conclusions

We propose a novel automated framework for thalamic and pulvinar parcellation that integrates MSMT-CSD derived fiber orientation distribution (FODs) with a spectral clustering algorithm stabilized by BIRCH initialization. By jointly leveraging voxel coordinates and spherical harmonic representations of diffusion profiles, the framework achieves robust, reproducible, and anatomically faithful segmentation of thalamus and pulvinar. Compared to baseline k-means, our method provides more stable subdivisions, including reliable recovery of the MD and partial identification of the LGN, with Dice reflecting spatial alignment with the Krauth-Morel atlas. These results demonstrate that spectral clustering of diffusion features yields biologically meaningful thalamic subdivisions and establishes a scalable foundation for future multimodal investigations.

## Supporting information

S1 File Supplementary Materials. Additional figures and tables supporting the results presented in the manuscript.
(DOCX)
