# Supplementary material for: Robust thalamic nuclei segmentation using spectral clustering of fiber orientation distributions
Source: PLoS One. 2026 Mar 25;21(3):e0345649. doi: 10.1371/journal.pone.0345649 (PMC13016311; doi:10.1371/journal.pone.0345649)
Supplement: S1 File — Additional figures and tables supporting the results presented in the manuscript. (DOCX) [file pone.0345649.s001.docx]

# **Thalamic Parcellation**

## **Atlas-Based Interpretation of Clustering Results**

S1 Table summarizes the correspondence between thalamic nuclei from the Krauth–Morel and Allen Brain Human atlases and the cluster labels obtained from spectral clustering and k-means, while also revealing how the reference atlases themselves are constructed through merged composite nuclei. While there is no one-to-one correspondence between the clustering solutions and reference atlas, several consistent patterns emerge across the data.

The anterior ventral nuclei (AV and VA) consistently map to a single cluster (cluster 1) in both clustering approaches, suggesting these regions share a common microstructural signature that is not separable at the current resolution. Similarly, the medial nuclei MD-Pf and CM both map to cluster 2 regardless of clustering method, indicating a stable and dominant diffusion profile in this region. The ventral lateral and ventral posterior nuclei show more variable correspondences, with VLa mapping to clusters 8 or 7, VLP either remaining unified (cluster 9) or fragmenting (clusters 4,7), and VPL mapping to clusters 7 or 6. The pulvinar demonstrates notable heterogeneity, subdividing into either two clusters (3,5) or three clusters (3,4,5) depending on the method, reflecting the known anatomical complexity of this region. Small sensory nuclei like LGN appear in one solution (cluster 6) for SC but not the other, while MGN is absent from both clustering solutions, possibly due to size or signal characteristics at the current resolution.

**S1 Table.** Correspondence Between Reference Thalamic Nuclei and Clustering-Derived Labels


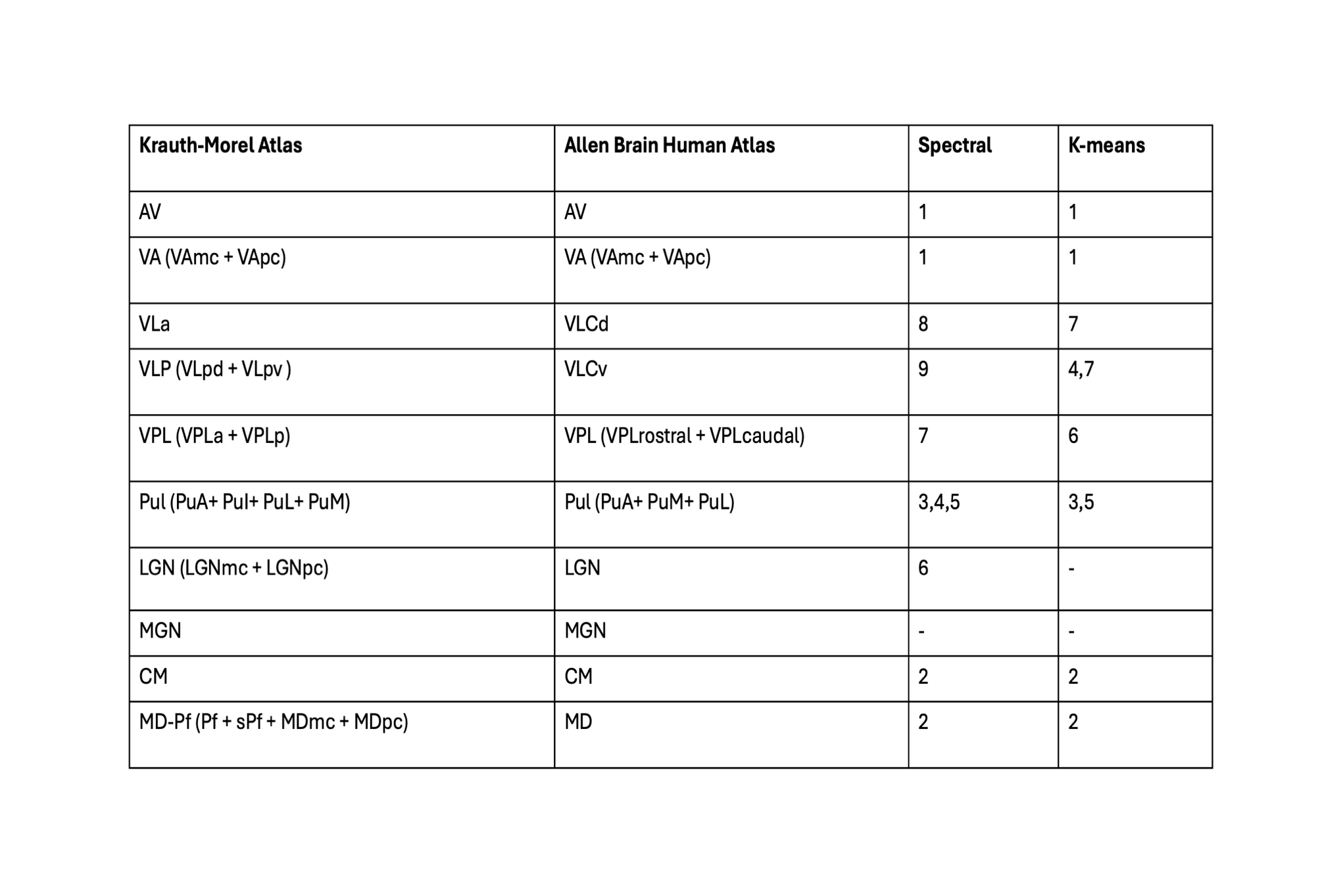


## **Effect of BIRCH pre-clustering**

We evaluated spectral clustering both with and without the BIRCH pre-clustering step. As shown in S1 Fig, spectral clustering without BIRCH was only somewhat stable at eight clusters, while the nine-cluster solution appeared random. The instabilities at eight clusters are evident in the spatial probabilistic label maps, particularly in the ventral lateral region where boundary fuzziness increased, indicating greater inter-subject boundary overlap. In contrast, incorporating BIRCH as a pre-clustering step yields more compact nuclei and sharper probabilistic boundaries, reflecting improved stability and reproducibility.


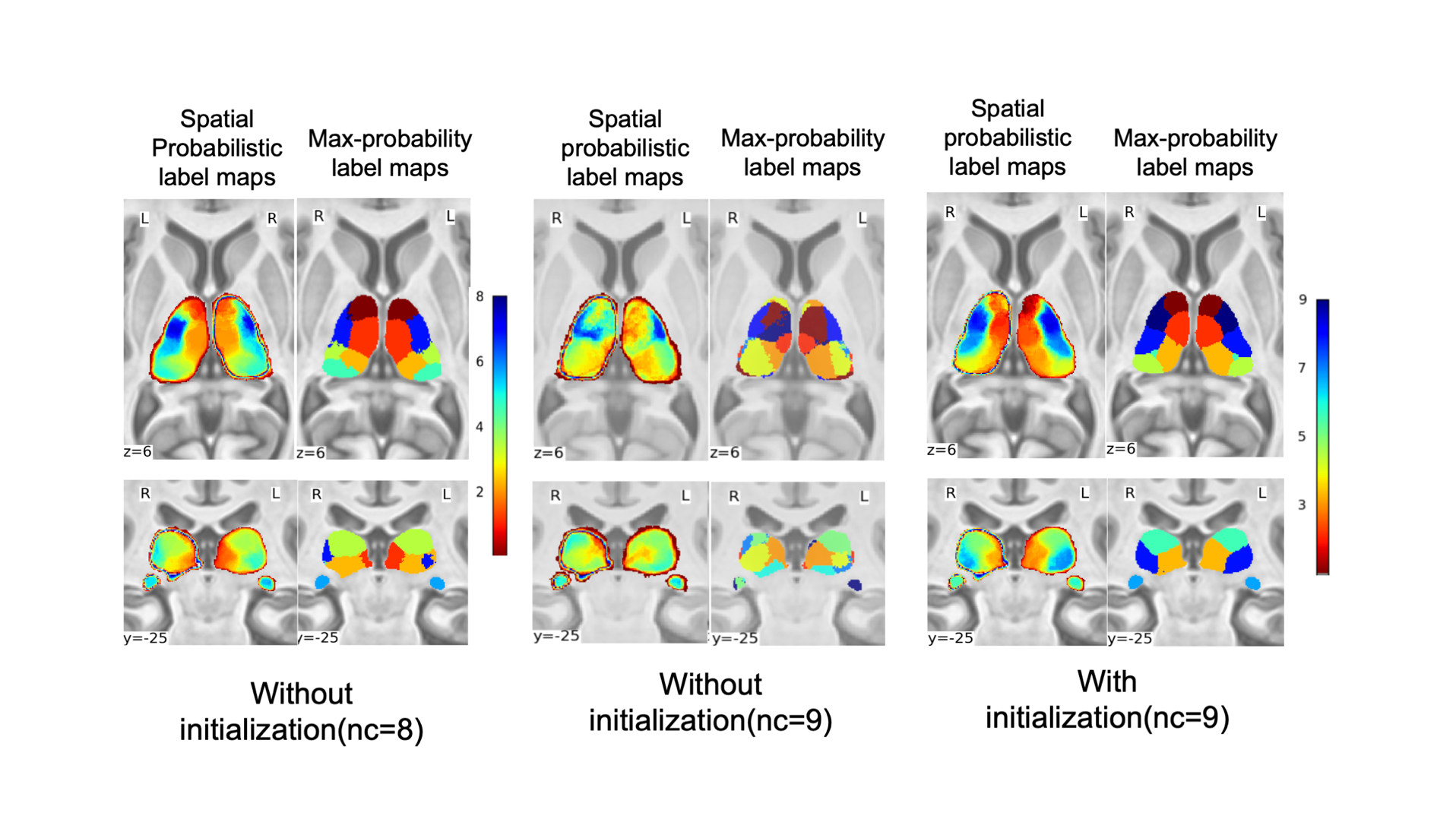


**S1 Fig. Effect of Initialization on Spectral Clustering for Thalamus Parcellation.** Comparison of spectral clustering with and without initialization for thalamus parcellation. Results are shown for clustering into 8 and 9 subdivisions without initialization (left and middle panels), and 9 subdivisions with initialization (right panel). For each condition, the group-level maximum probability maps (right columns) and spatial probability maps (left columns) are displayed. Overlays are shown on the ICBM 2009b T1w MNI template at axial slice z = 6mm and coronal slice y = –25mm.

## **Effect of** $\boldsymbol{\gamma}$ **parameter**

Keeping the $\alpha$ at 0.5 based on Battistella’s work, we examined a range of $\gamma$ values and evaluated their effect on thalamic parcellation using maximum probability maps thresholded at 50%, generated using 5 subjects. As shown in S2a Fig, $\gamma$ values in the range 32–64 show less inter-subject variability in the spatial organization of thalamic nuclei, with fewer unlabeled voxels, indicating greater agreement across subjects. Based on this observation, this interval was fixed as the operating range for the thalamus. Within this range, $\gamma$ was selected by minimizing the mean absolute error between the $D_{vox}$ and $D_{fods}$, resulting in $\gamma$ = 48 (S2b Fig). ${D_{vox}=\parallel c_{i}-c_{j}\parallel}_{2}$ and $D_{fods}={\parallel f_{i}-f_{j}\parallel}_{2}$ as defined in Equation 1(Material and Methods).


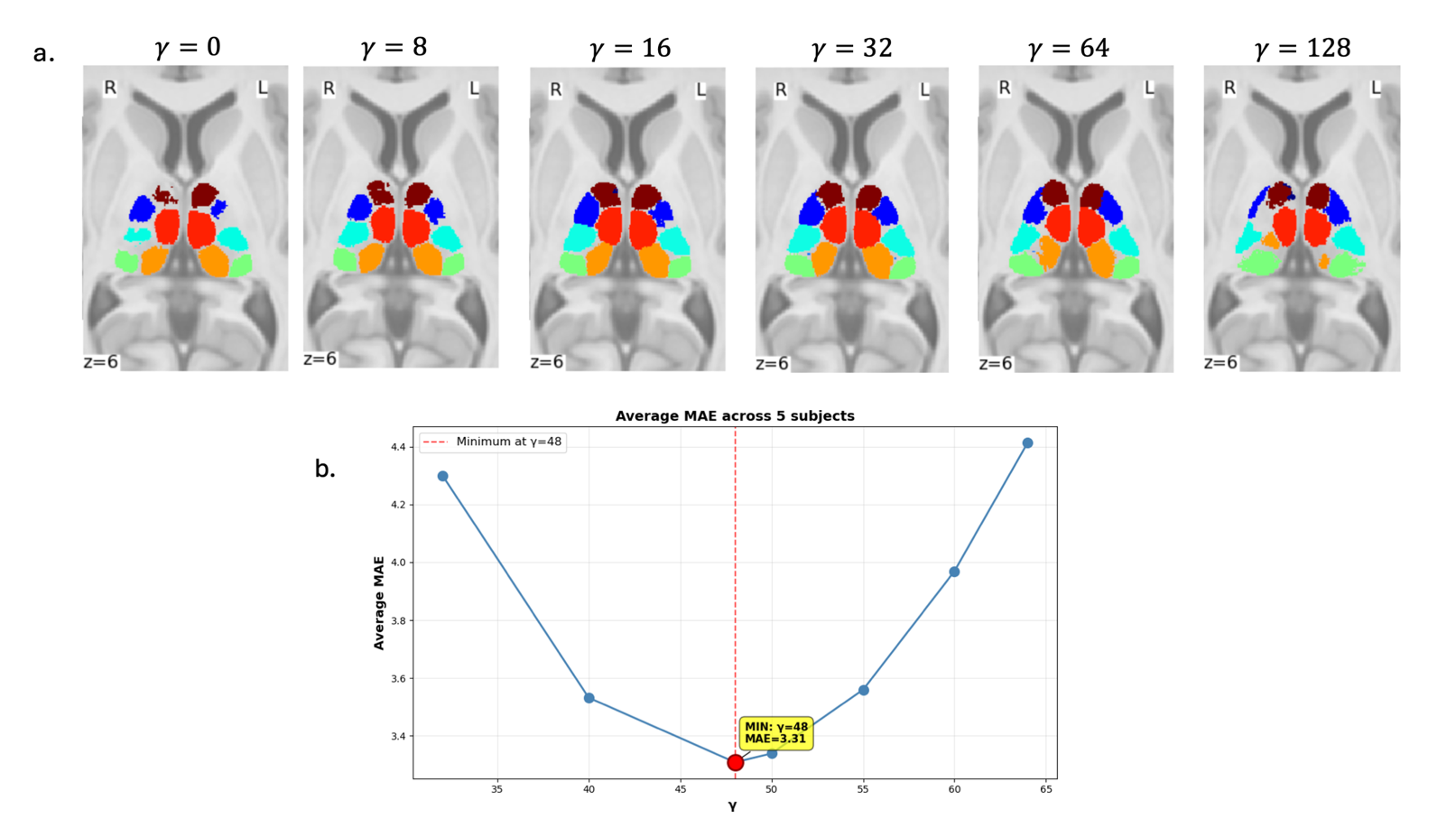


**S2 Figure: Gamma Tuning for Thalamic Clustering.** (a) Maximum probability label maps (thresholded at 50%) for 5 subjects, showing thalamic parcellation sensitivity to γ parameter. (b) Mean absolute error between $D_{vox}$ and $D_{fods}$ across $\gamma$ values, with optimal selected at minimum MAE.

## **Sensitivity to** $\boldsymbol{\alpha}$ **parameter**

To study the effect of the weighting parameter $\alpha$ on thalamic parcellation, we performed spectral clustering on the 30 subjects using three weighting schemes: $\alpha$ = 1 (spatial information only), $\alpha$ = 0.5 (equal weighting of spatial and diffusion features), and $\alpha$ = 0 (diffusion information only) keeping $\gamma$ at 48. Supplementary Figure 3 shows the corresponding group-level thalamus segmentation results, with the top row displaying maximum probability maps and the bottom row showing probability maps thresholded at 50%. In the thresholded maps, voxels without assigned labels indicate reduced agreement across subjects and therefore lower inter-subject stability. Both extreme settings ($\alpha$ = 1 and $\alpha$ = 0) exhibit larger regions of unlabeled voxels. In contrast, $\alpha$ = 0.5 yields fewer unlabeled voxels and more spatially coherent thalamic nuclei, indicating improved inter-subject stability. Given the homogeneity of the dataset, reduced inter-subject variability and correspondingly consistent label assignments across subjects are expected.


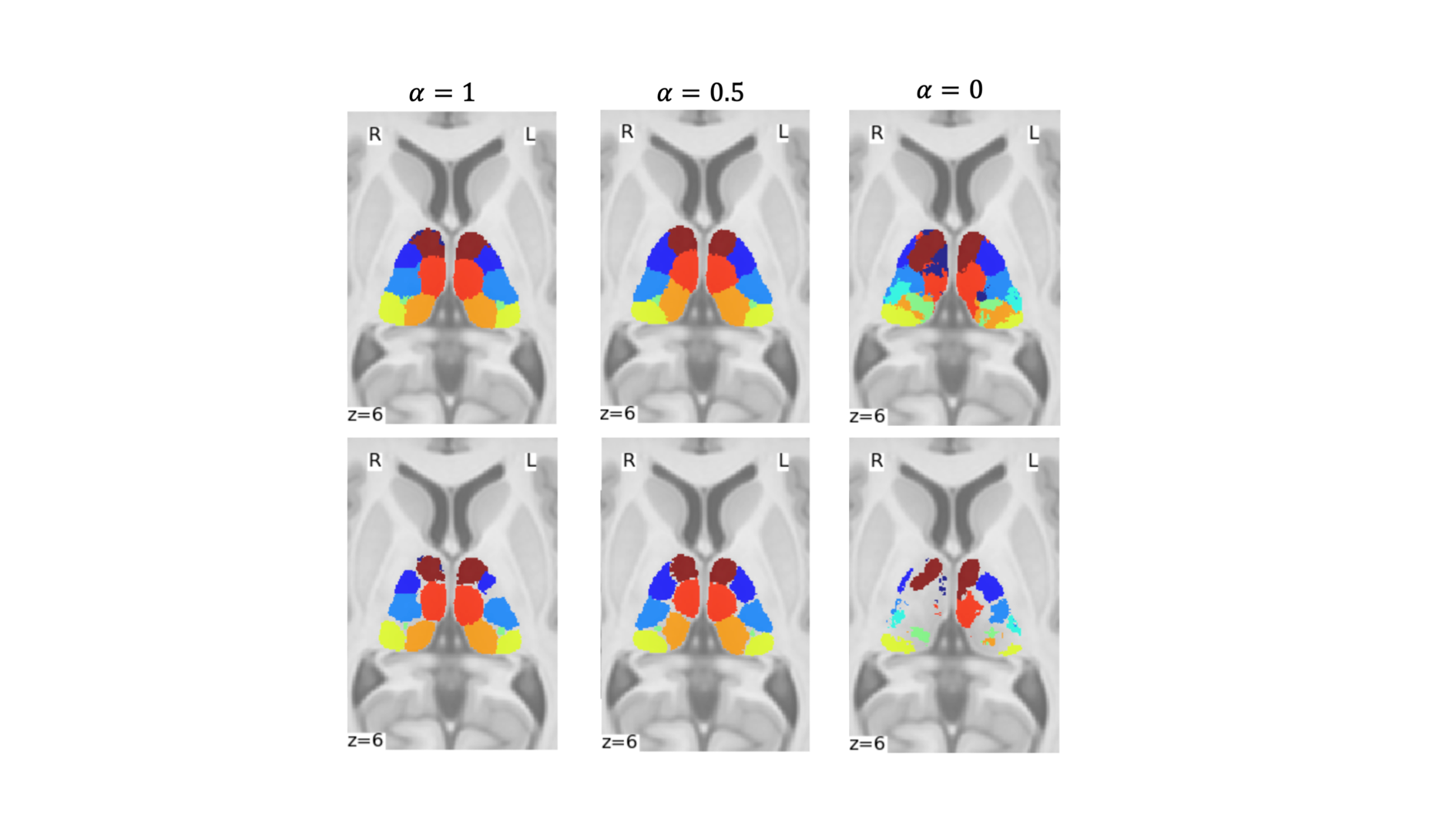


**S3 Fig. Sensitivity Analysis of the α Parameter in Thalamic Clustering.** Group-level thalamus segmentation results under different weighting schemes between spatial ($\alpha$ = 1, structural information only) and diffusion features ($\alpha$ = 0, diffusion information only). The top row shows maximum probability maps, and the bottom row shows thresholded probability maps at 50%.

## **K-means Cluster Cardinality**

S4 Fig indicates that k-means (k=7) performs better than k-means (k=9). We have also established that spectral clustering performs better than k-means (k=7) across many nuclei and can handle addition of LGN and MGN. Taken together, this implies spectral clustering performs better than k-means (k=9).

##
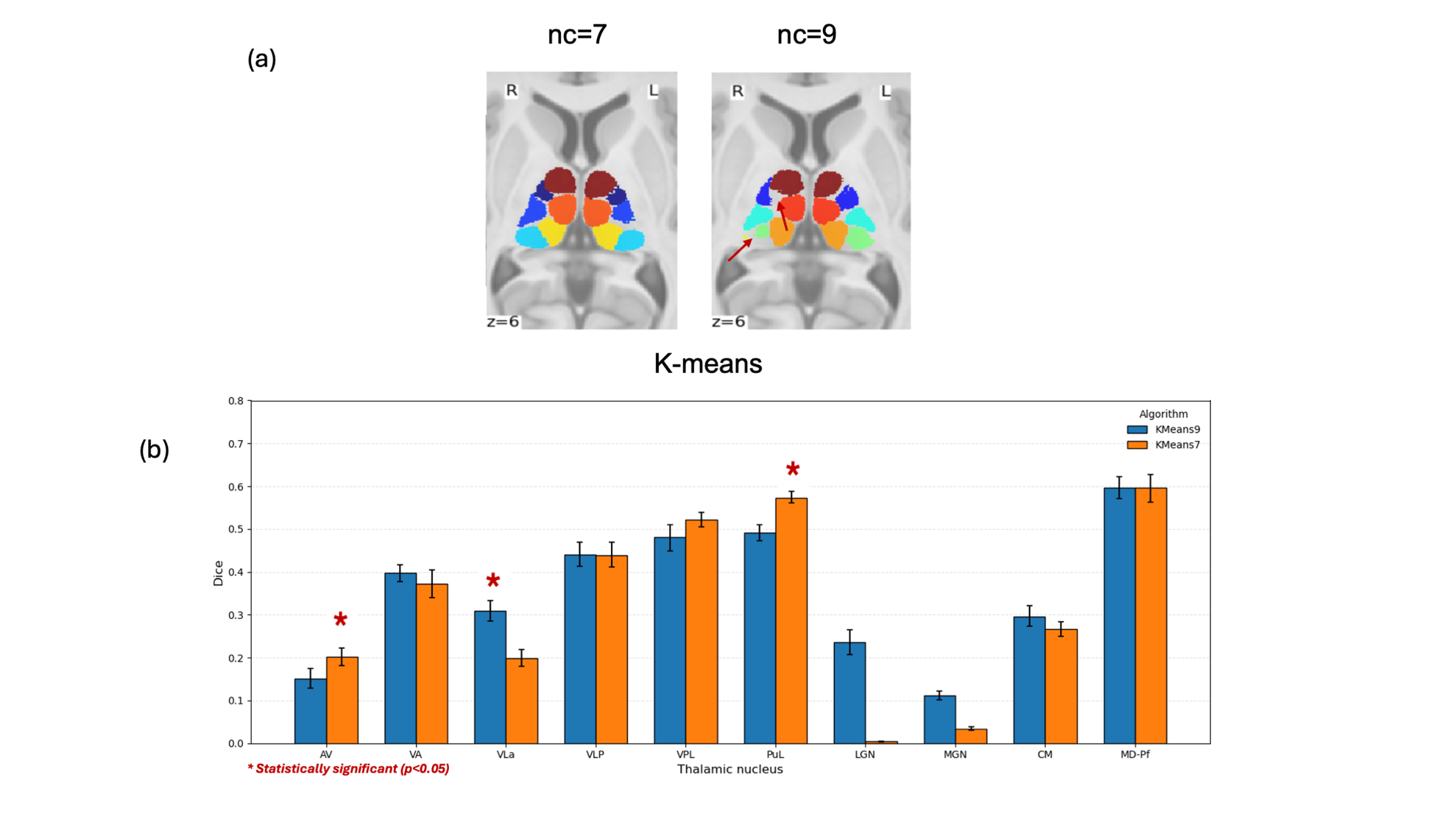


**S4 Fig. Group-Level Thalamus Segmentation Using k-means.** (a) Group-level thalamic segmentation probability label maps obtained using k-means clustering (nc = 7 and nc = 9), displayed at a 50% probability threshold. (b) Bar plots of Dice similarity scores across thalamic nuclei for k-means (nc = 7, nc = 9). Statistically significant differences are indicated by red stars.

## **Dice Score with Saranathan Atas as reference**

S5 Fig shows Dice-based confusion matrices comparing data-driven thalamic segmentations with Saranathan Atlas. Results are shown for k-means clustering with 7 and for spectral clustering with 9 clusters. Both methods show similar overall patterns, with AV, VA, and MD–Pf consistently mapping to the same dominant clusters.
The pulvinar is split across multiple clusters in both cases, indicating comparable heterogeneity.


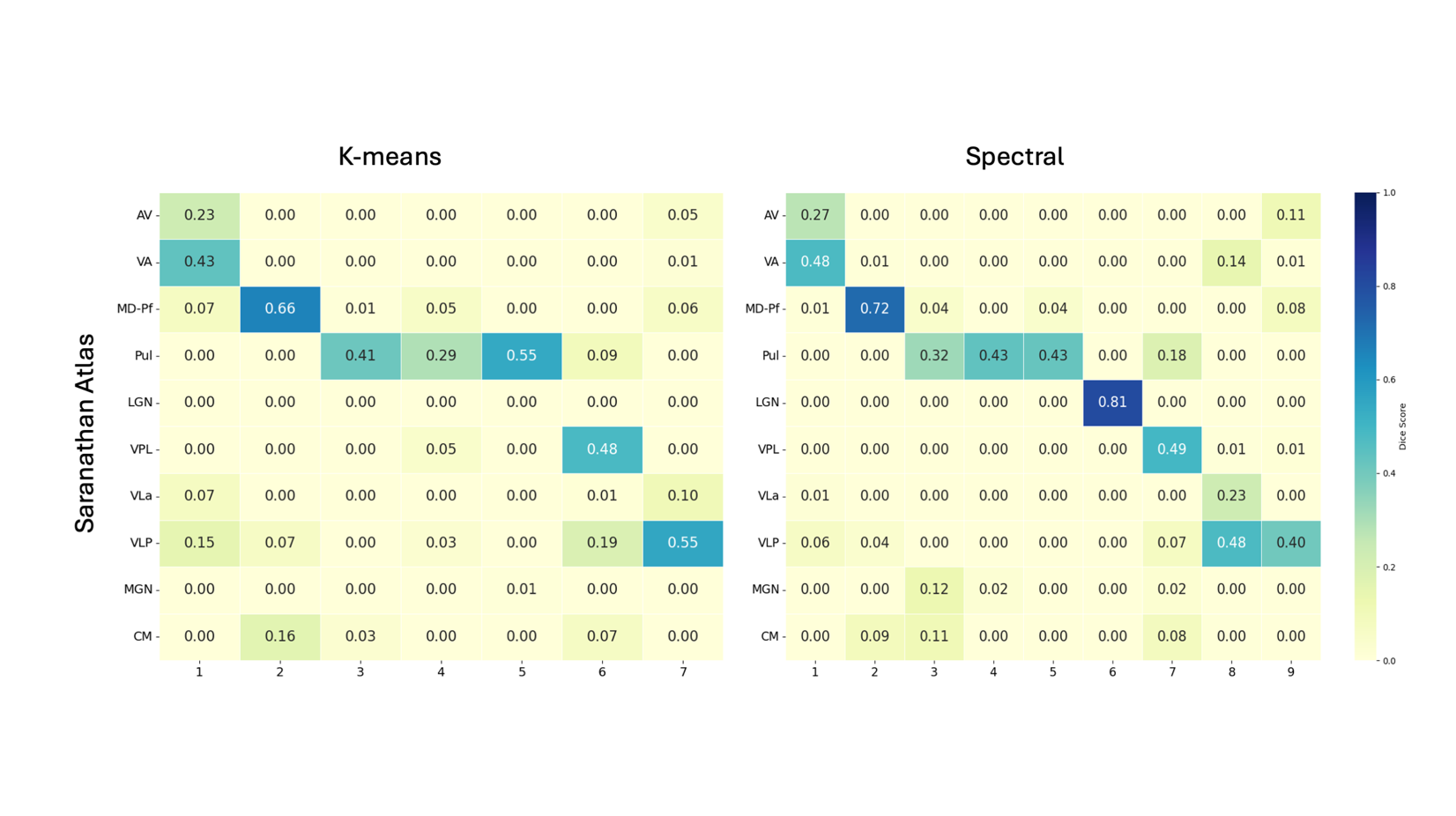


**S5 Fig. Evaluation of thalamic segmentation using confusion matrices.** Comparing Saranathan atlas with the maximum‐probability maps generated by each clustering method, K-means(nc=7) and spectral(nc=9) quantified via Dice coefficients

## **Subject-Level Visualization of Thalamic and Pulvinar Segmentations**

The five subjects shown in S6 Fig were selected for qualitative evaluation during $\gamma$ parameter tuning and for examining subject-level connectivity patterns for pulvinar. These examples illustrate the consistency of the segmentation results across individuals and provide visual insight.


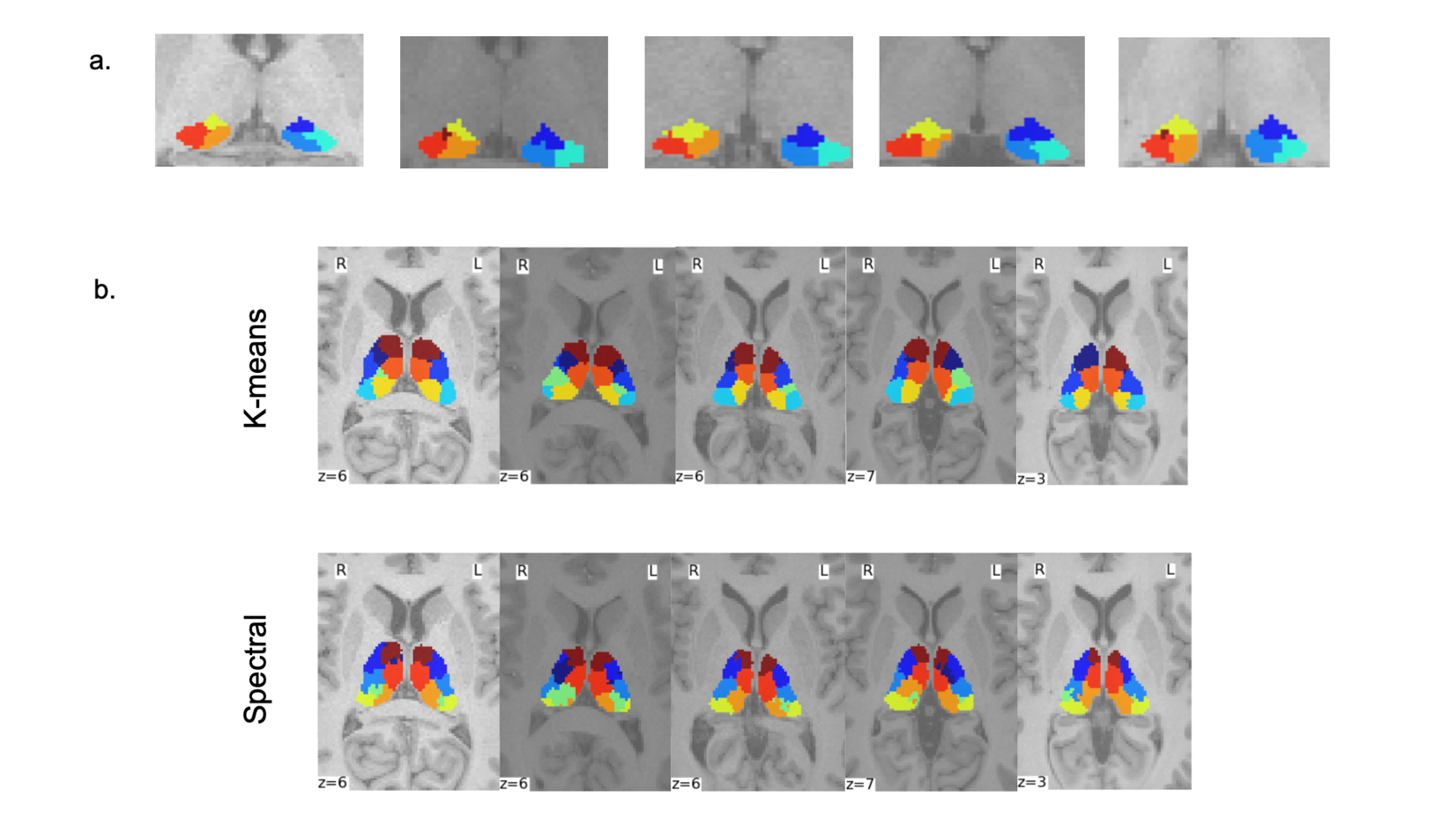


**S6 Fig. Segmentation Results for Pulvinar and Thalamic Nuclei**. (a)Pulvinar and (b)Thalamic nuclei segmentation results for five subjects. Axial slices show results from modified spectral clustering and K-means, overlaid on each subject’s T1w image.

Pulvinar Subdivision:

## Sensitivity to $\gamma$ parameter:

With $\alpha$ fixed at 0.5, we assessed the impact of varying γ on pulvinar segmentation by examining maximum probability maps thresholded at 50%, similar to thalamus. Supplementary Figure 6a shows that $\gamma$ values between 16 and 64 less intersubject variability of pulvinar subdivisions across subjects. Within this range, $\gamma$ was selected by minimizing the mean absolute error between the $D_{vox}$ and $D_{fods}$, resulting in $\gamma$ = 48 (S2b Fig). ${D_{vox}=\parallel c_{i}-c_{j}\parallel}_{2}$ and $D_{fods}={\parallel f_{i}-f_{j}\parallel}_{2}$ as defined in Equation 1(Material and Methods).


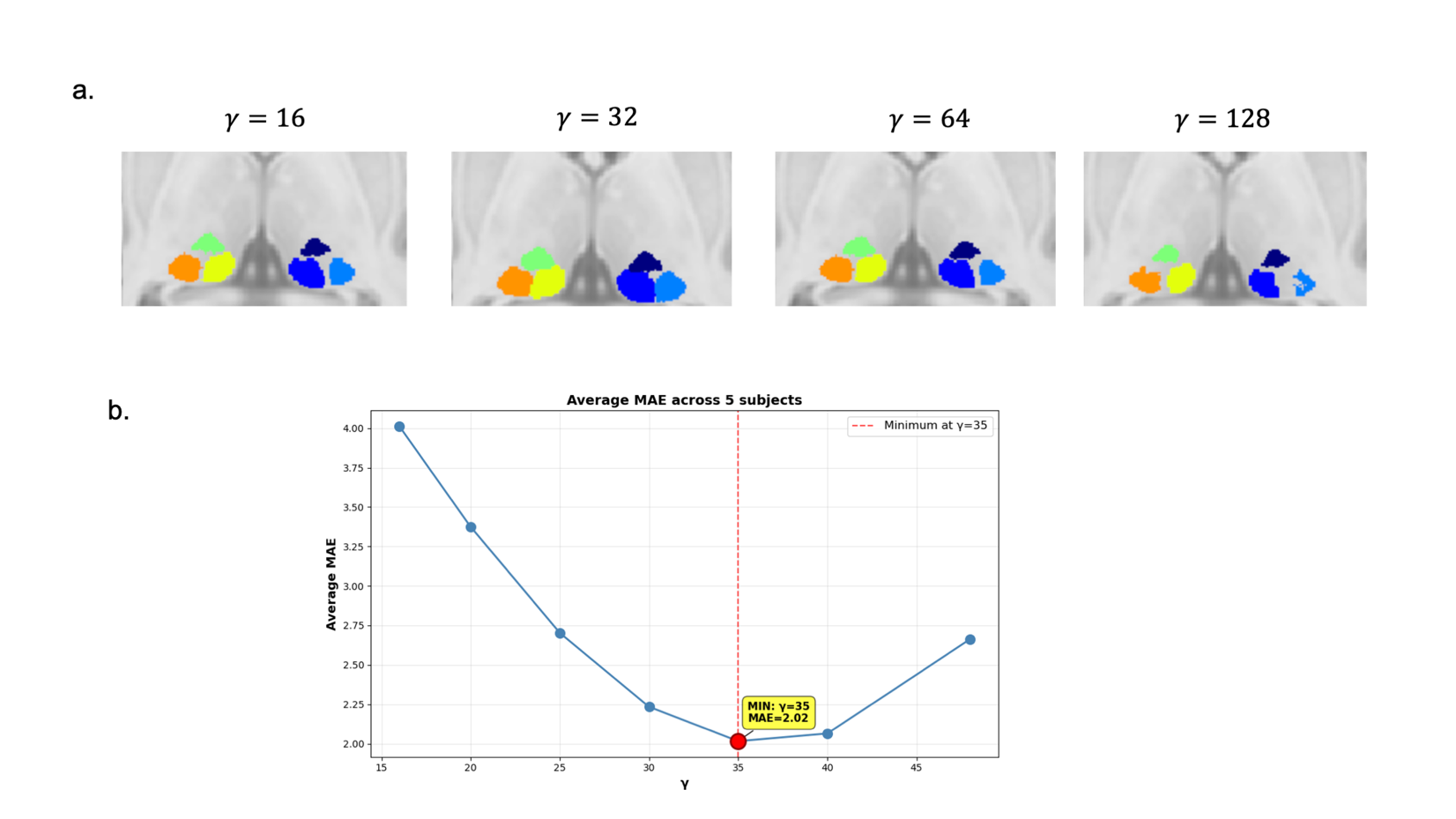


**S7 Fig. Gamma Tuning for Pulvinar Clustering.** (a) Maximum probability label maps (thresholded at 50%) for 5 subjects, showing pulvinar parcellation sensitivity to γ parameter. (b) Mean absolute error between $D_{vox}$ and $D_{fods}$ across $\gamma$ values, with optimal selected at minimum MAE.

## Evaluation of pulvinar parcellation stability:

As shown in S8a Fig., without BIRCH initialization the pulvinar could only be partitioned into three broad groups (PuM-1, PuM+PuA, and PuL+PuI), whereas with initialization we get 4 distinct clusters. S8b Fig. summarizes quantitative properties of the clusters across subjects. It shows that cluster volumes ratio is broadly comparable, with moderate variability, and that cluster centroids are positioned at similar distances from the pulvinar boundary, indicating consistent spatial placement.

**
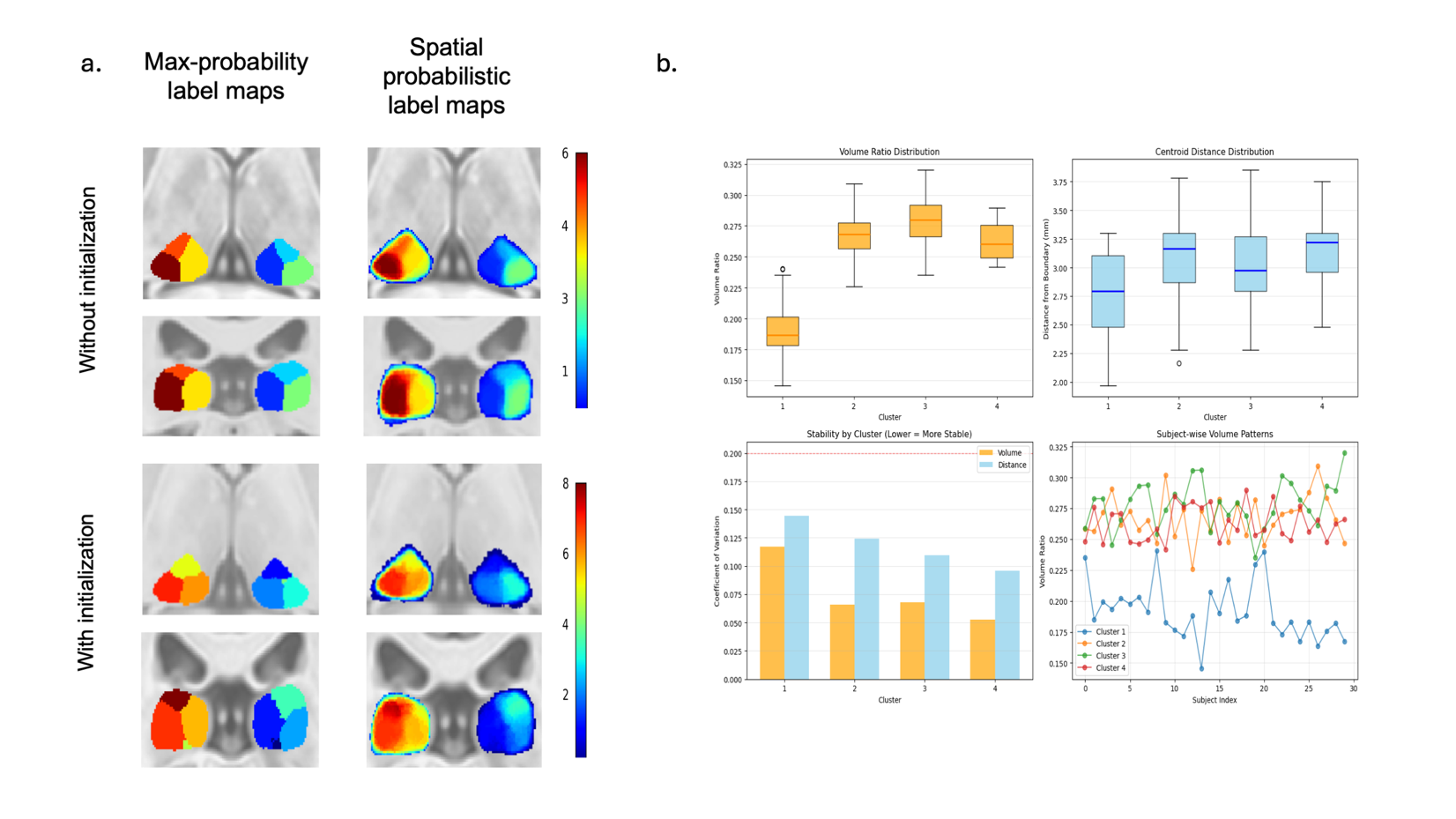
**

**S8 Fig. Effect of Initialization on Pulvinar Spectral Clustering and Inter-Subject Variability.** (a)Comparison of spectral clustering with and without initialization for pulvinar parcellation. Results are shown for clustering into 3 subdivisions without initialization, and 4 subdivisions with initialization. For each condition, the group-level maximum probability maps and spatial probability maps are displayed. Overlays are shown on the MNI152 template at coronal slice z = 6. (b) A statistical analysis of the differences between subjects in pulvinar segmentation using spectral clustering

## Structural Connectivity of Pulvinar Subdivisions From K-means clustering:

If we look at the connectivity of the Krauth-Morel atlas with cortical and subcortical region, we observe some known connections. We found that the anterior pulvinar (PuA) subdivisions connect with motor and somatosensory cortical regions. Lateral pulvinar (PuL) subdivisions show predominant connectivity with visual cortices, aligning with established roles in visual attention and integration. Medial pulvinar (PuM) subdivisions demonstrate strong coupling with limbic structures, particularly the amygdala and hippocampus. In contrast, inferior pulvinar (PuI) subdivisions exhibit more localized and constrained connectivity, mainly targeting visual regions with limited engagement of other cortical areas.


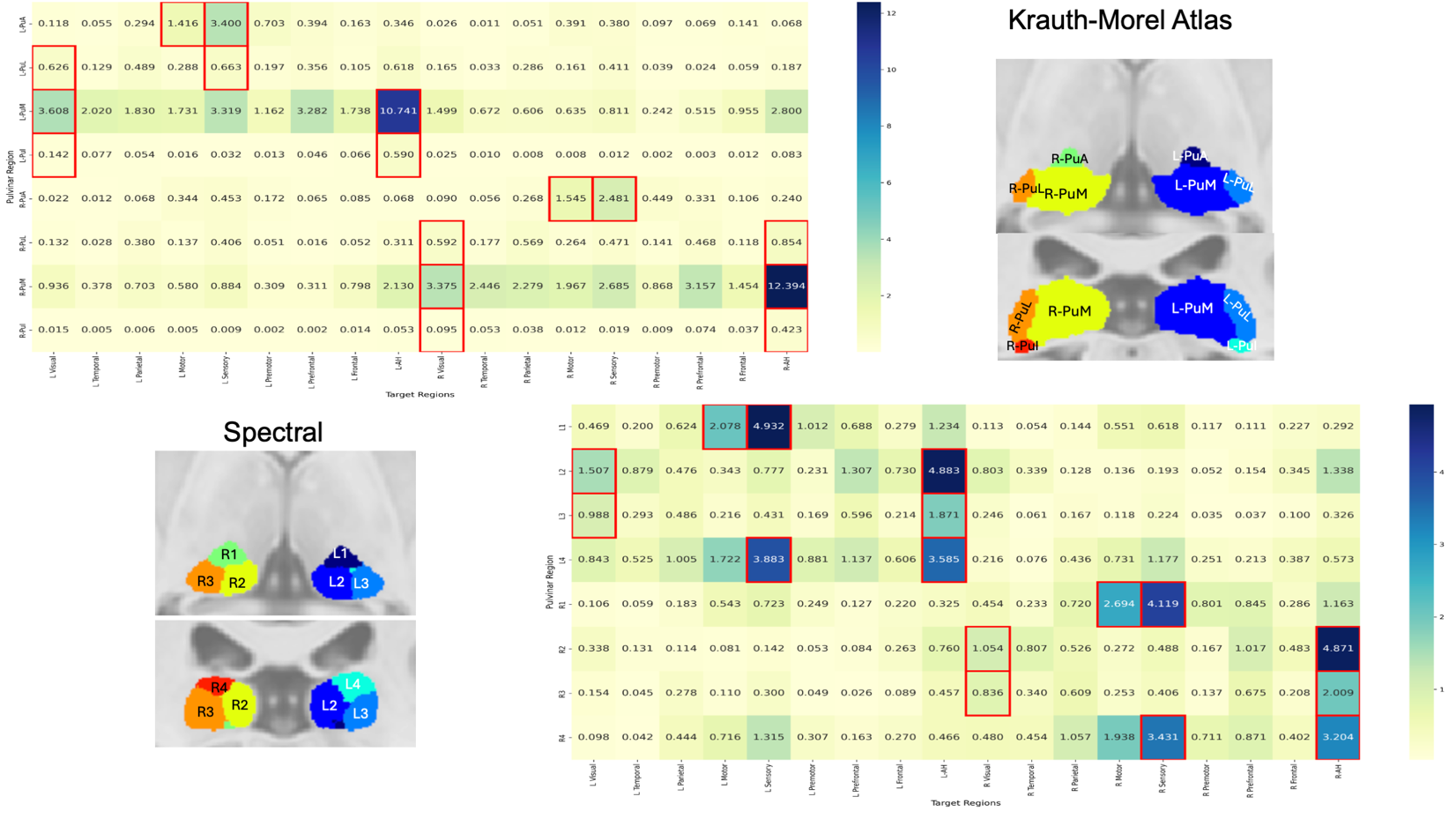


**S9 Fig. Pulvinar–Cortical Connectivity Patterns and Parcellation Visualization.** Heatmaps illustrate the connection strengths of pulvinar subdivisions with cortical regions, while axial overlays show pulvinar parcellations on the ICBM 2009b T1w MNI template. Kauth-morel atlas-based subdivisions (on top) and spectral clustering based pulvinar parcellation (at bottom).
