# Supplementary material for: Robust thalamic nuclei segmentation using spectral clustering of fiber orientation distributions
Source: PLoS One. 2026 Mar 25;21(3):e0345649. doi: 10.1371/journal.pone.0345649 (PMC13016311; doi:10.1371/journal.pone.0345649)
Supplement: Attachment — Submitted filename: Response_to_Reviewers.docx [file pone.0345649.s003.docx]

**Reviewer #1:**

We sincerely thank the reviewer for the thorough and constructive review, which substantially helped improve the clarity and rigor of the manuscript.

We would like to clarify that the primary aim of this work is to propose an improvement over the framework of k-means clusetering on fiber orientation distribution (FOD)-derived features introduced by Battistella et al., by using a spectral clustering scheme and further applying it to subdividing the pulvinar nucleus which is an important target for emerging epilepsy DBS applications.

We acknowledge the reviewer’s observation regarding the absence of explicit statistical testing in the initial submission. In response, we have revised the manuscript to better articulate the comparative evaluation framework and to clarify the basis of our conclusions. As part of this revision, we report results for k-means clustering with both $nc=7$and $nc=9$ using the Krauth Morel atlas; among these, the $nc=9$configuration showed poorer agreement with reference atlas and reduced inter-subject stability. We have now expanded the thalamus study to include three reference atlases (Krauth Morel, Saranathan, and the Allen Brain Human Atlas). The comparative evaluation between k means with nc = 7 and spectral clustering was conducted across all three reference atlases.

In addition, we have expanded the analysis to explicitly include a dedicated k-means evaluation for the pulvinar, enabling a more direct and transparent comparison between methods. We believe these revisions address the reviewer’s concerns and strengthen the methodological positioning of the proposed approach.

All revisions in the manuscript have been explicitly labeled as R1CX(major/minor), where X indicates the comment number and editorial comments are labeled as EC.
**Major issues**

**1. Page 2, Line 20: How much does the approach improve stability and anatomical consistency by?**

Anatomical consistency in our study is quantified using Dice overlap, as reported in Table 1. However, these results show that Dice overlap is both nucleus-dependent and atlas-dependent, with spectral clustering yielding higher Dice for some nuclei and k-means for others.

For this reason, the abstract does not report a single aggregated improvement in anatomical consistency. Instead, it provides representative Dice values for major nuclei and highlights method-specific behavior (e.g., identification of smaller nuclei such as LGN), while the full per-nucleus and per-atlas statistical comparisons are presented in Table 1.

**2. Page 2, Line 23: Why does smoothness matter? How was it determined that the parcellations were “more biologically meaningful”?**

By “smoothness,” we refer to the consistency with which voxels are assigned to the same label across subjects, as reflected in group-level spatial probabilistic maps. A smoother parcellation is characterized by more gradual spatial variation in label probabilities and reduced voxel-level dispersion, indicating that neighboring voxels tend to share similar probability mass for a given label across subjects. By “biologically meaningful,” we referred to agreement with established thalamic anatomy, assessed using Dice overlap(which was higher for some nuclei and comparable for others), as well as the ability to identify small structures such as the LGN. We have revised the manuscript (**Page 2, Line 19**) to replace these qualitative terms with explicit, metric-based descriptions to improve clarity.

**3. Page 2, Line 24: How many subnuclei in total were evaluated?**

The Krauth-Morel atlas labels were consolidated to align with the 12-label Morel nomenclature implemented in the THOMAS algorithm, resulting in the evaluation of 12 major thalamic nuclei in total. The grouping of the reference atlases is shown in S1 Table.

**4. Page 3, Line 54: What about anatomical variability across atlases (Neudorfer et al., 2024;** [**https://doi.org/10.1016/j.neurot.2023.e00313**](https://nam11.safelinks.protection.outlook.com/?url=https%3A%2F%2Fdoi.org%2F10.1016%2Fj.neurot.2023.e00313&data=05%7C02%7Cdebottamad%40arizona.edu%7Cbd6f4ed52fd646cd3e7408de36803743%7C5ee35505eb8e4929937d645df5013288%7C1%7C0%7C639008121481484667%7CUnknown%7CTWFpbGZsb3d8eyJFbXB0eU1hcGkiOnRydWUsIlYiOiIwLjAuMDAwMCIsIlAiOiJXaW4zMiIsIkFOIjoiTWFpbCIsIldUIjoyfQ%3D%3D%7C0%7C%7C%7C&sdata=r3pdvVTytmyb2UvN6IYN4aOabSys2sMxkdy3a4Qme4Q%3D&reserved=0)**)?**

We acknowledge the substantial anatomical variability across thalamic atlases highlighted by Neudorfer et al. (2024). We will like to note that Behrens et al. (Reference 3) explicitly describe connectivity-based thalamic parcellations as differing from cytoarchitectonic atlases such as the Morel atlas. Our focus is on comparing FOD based clustering strategies rather than evaluating fiber-tracking based segmentation approaches; therefore, a detailed analysis of tractography-based parcellations is beyond the scope of the present study. We modified the manuscript (**Page 4, Line 54**) to clarify this point.

**5. Materials and Methods: Why is there no statistical analysis section?**

We have now added a dedicated statistical analysis subsection within the Materials and Methods section and Table 1 in the results to address the concerns.

**6. Page 6, Line 101: How many subjects in total are in the HCP Young Adult database? If more than 30, why were only 30 subjects included in this study?**

The HCP cohort is highly homogeneous, comprising 1200 young healthy adults. We used 30 subjects as it is common practice in the literature to draw randomly selected subsets from this dataset rather than analyzing the full cohort. We have clarified this point in the manuscript accordingly (**Page 6, Line 101**) to reflect this point.

**7. Page 7, Line 133: Because it “forms the primary methodological contribution”, what exactly was modified in the spectral clustering framework? The initialization (via BIRCH clustering), the features used (FOD-based), the thalamus mask generation?**

Our primary methodological contribution is the incorporation of a BIRCH-based initialization step into a conventional spectral clustering pipeline. Specifically, we use BIRCH to obtain stable initial cluster assignments, which are then used to initialize the spectral clustering procedure. We have revised the manuscript accordingly (**Page 7, Line 139**) to clarify this point.

**8. Page 7, Line 136: How many superclusters are generated? Is that determined in advance?**

Superclusters are generated using a distance-based threshold within the BIRCH initialization stage. We set the distance threshold to 1, as this value provided a practical balance between computational efficiency and clustering stability across both segmentation tasks. With this setting, the framework typically produces approximately 500 superclusters for whole-thalamus parcellation and around 100 superclusters for pulvinar-specific segmentation. Changes are made in the manuscript (**Page 8, Line 143**) to reflect the idea.

**9. Page 7, Line 141: Seeding does not have to be random for k-means clustering in this context. For example, it can be informed by an atlas (Malaga et al., 2023;** [**https://doi.org/10.1016/j.neurom.2022.09.013**](https://nam11.safelinks.protection.outlook.com/?url=https%3A%2F%2Fdoi.org%2F10.1016%2Fj.neurom.2022.09.013&data=05%7C02%7Cdebottamad%40arizona.edu%7Cbd6f4ed52fd646cd3e7408de36803743%7C5ee35505eb8e4929937d645df5013288%7C1%7C0%7C639008121481502455%7CUnknown%7CTWFpbGZsb3d8eyJFbXB0eU1hcGkiOnRydWUsIlYiOiIwLjAuMDAwMCIsIlAiOiJXaW4zMiIsIkFOIjoiTWFpbCIsIldUIjoyfQ%3D%3D%7C0%7C%7C%7C&sdata=6pdq%2FOfsHZPnIBXrD299wN7DizSgq4zhlmJZauwfarI%3D&reserved=0)**).**

We agree that k-means clustering does not inherently require random seeding and that atlas-informed initialization strategies, such as those described by Malaga et al. (2023), represent a viable alternative in this context. In the revised manuscript (**Page 8, Line 168** ),we have clarified our wording to avoid implying that random initialization is intrinsic to k-means.

**10. Page 8, Line 159: For the k-means clustering comparison, were the same features used?**

Yes, the same set of features was used for the k-means clustering analysis. We have revised the manuscript (**Page 9, Line 176**) to clearly reflect this.

**11. Page 8, Line 160: Which subnuclei did the seven clusters represent? Based on which atlas?**
Spectral clustering with nine clusters resulted in: Cluster 1 corresponding to AV+VA, Cluster 2 to MD–Pf, Cluster 3 to Pu1+CM, Cluster 4 to Pu2, Cluster 5 to Pu3, Cluster 6 to LGN, Cluster 7 to VPL, Cluster 8 to VL, and Cluster 9 to VLP. We have added this information to the S1 Table.

**12. Page 8, Line 161: Why not use nine clusters for k-means clustering? Is it a fair comparison if spectral clustering used nine clusters, while k-means clustering used seven?**

We evaluated both seven and nine clusters for k-means to ensure a fair comparison. As discussed in the newly added Results subsection on k-means cluster cardinality, the Adjusted Rand Index (ARI) analysis demonstrates that k-means performance degrades substantially when increasing the number of clusters from seven to nine (p < 0.001). This finding is further supported by Dice as shown in S4 Fig. Based on this evidence, using seven clusters for k-means and nine clusters for spectral clustering reflects a balanced and fair comparison. This is also consistent with Battistella’s finding of 7 being the optimal number of clusters for stability.

**13. Page 9, Line 167: Why specifically use four clusters? Which anatomical regions do they represent?**

The number of clusters was fixed at four based on evidence from recent studies, References 25 and 26, and the histology-based Morel atlas. We have made changes in the manuscript (**Page 10, Line 187**) to reflect this clearly.

**14. Page 9, Line 174: How many subnuclei are in this atlas? (14 based on Figure 4?) Which subnuclei?**

Krauth-Morel labels were grouped to align with the 12-label Morel nomenclature used by the THOMAS algorithm, thereby facilitating the analysis of major thalamic nuclei. The grouping of the reference atlases is shown in S1 Table.

**15. Page 9, Line 175: What does “visually assess cluster consistency” mean? What exactly was being looked at? How was it determined if the clustering looked reasonable? Consistency is not the same as (anatomical) accuracy.**

By “visually assess cluster consistency,” we refer to qualitative inspection of group-level probability maps to evaluate inter-subject stability of clustering . This assessment was used to evaluate inter-subject stability and reproducibility, not anatomical accuracy. Anatomical agreement was evaluated separately using atlas-based overlap metrics (e.g., Dice). Given the highly homogeneous nature of the dataset, such visual assessment is appropriate for assessing consistency. We have revised the manuscript (**Page 10, Line 195**) to clarify this distinction.

**16. Page 9, Line 183: Why was no similar quantitative analysis using Dice scores performed for pulvinar clustering?**

A quantitative Dice-based analysis for pulvinar clustering has now been added and shown in Fig 6.

**17. Page 10, Line 188: Guedj and Vuilleumier, 2020 (**[**https://doi.org/10.1016/j.neuroimage.2020.117162;**](https://nam11.safelinks.protection.outlook.com/?url=https%3A%2F%2Fdoi.org%2F10.1016%2Fj.neuroimage.2020.117162%3B&data=05%7C02%7Cdebottamad%40arizona.edu%7Cbd6f4ed52fd646cd3e7408de36803743%7C5ee35505eb8e4929937d645df5013288%7C1%7C0%7C639008121481519642%7CUnknown%7CTWFpbGZsb3d8eyJFbXB0eU1hcGkiOnRydWUsIlYiOiIwLjAuMDAwMCIsIlAiOiJXaW4zMiIsIkFOIjoiTWFpbCIsIldUIjoyfQ%3D%3D%7C0%7C%7C%7C&sdata=bfC4Ks8N2CUCgSR4ChrgHvG9p4Y109FUaEDGThROESk%3D&reserved=0) **Reference 26) found five pulvinar clusters, so why use four in this study?**

Although Guedj and Vuilleumier (2020) reported five pulvinar clusters, more recent connectivity-informed studies (References 25 and 26) consistently adopt a four-cluster subdivision. Our use of four clusters aligns with this contemporary literature and with the Morel atlas. We have added this clarification to the manuscript (**Page 10, Line 187).**

**18. Page 10, Line 200: For k-means clustering, were the seeds randomly initialized, as opposed to being atlas-informed?**

The k-means clustering was initialized using a data-driven strategy involving 5,000 iterations to identify optimal starting centroids. This approach was adopted to minimize the influence of random seeding and follows the procedure described by Batistella et al. We have revised the manuscript to clarify the initialization process (**Page 9, Line 177**).

**19. Page 10, Line 205: Figure 4 highlights the consistency of spectral clustering across subjects; however, k-means clustering also appears consistent. This should be discussed as spectral clustering is not clearly better than k-means clustering based on this specific result.**

We now clarify that k-means clustering also shows intersubject consistency **when the LGN and MGN are removed from the thalamus mask**. This has been stated explicitly(**Page 15, Line 300**) to ensure a fair and transparent comparison with the spectral method.

**20. Page 10, Line 207: Similar to Comment 19, consistent parcellations do not appear to be unique to spectral clustering based on Fig. S3.**

We have removed Figure S3, as it did not provide additional information beyond the main results. In the revised manuscript as mentioned in Comment 19, we now acknowledge that both k-means (nc = 7) and spectral clustering (nc = 9) yield intersubject-consistent parcellations but k-means can only do that when the LGN and MGN are excluded from the thalamus mask.

**21. Figure 3: Why not show segmentation results for seven clusters using spectral clustering as well?**

As described in the **Materials and Methods (Page 9, Line 178)**, Battistella’s optimal seven-cluster solution excluded the LGN and MGN from the thalamus definition, whereas our thalamus mask (1-THALAMUS from THOMAS) explicitly includes these nuclei. Given this difference, and the fact that our reference framework comprises ten nuclei, a seven-cluster solution would not be well aligned with the anatomical scope of our analysis. For this reason, we focus on nine clusters.
**22. Figure 5b: What are the subnucleus-number pairs? For example, is VA equivalent to 1? Also, why are 10 subnuclei from the atlas evaluated against seven clusters for k-means clustering and nine clusters for spectral clustering? Is this a fair comparison?**

We have added a table in the supplement (S1 Table) which shows the cluster correspondence with reference atlases. Clusters are comprised of groups of nuclei in some cases and so yes, it is fair to compare. Since we are showing a heat map, it doesn not matter and we can detect if a cluster maps to multiple nuclei.

**23. Page 11, Line 225: Did spectral clustering produce significantly higher Dice scores for all key thalamic nuclei? This sentence as written suggests so. However, there were instances where k-means clustering performed better.**

We now clarify that Dice scores are dependent on the reference atlas and exhibit nucleus-specific differences between spectral clustering and k-means.The mauscript has been updated to avoid implying uniformly superior performance of spectral clustering across all nuclei (**Page 16, Line 360**).

**24. Page 11, Line 228: Given that k-means clustering was limited to seven clusters, was it possible for it to partially recover LGN? Again, is this a fair comparison against spectral clustering? (Partially addressed on Page 12, Line 242.)**

We added a dedicated Results subsection examining the effect of k-means cluster cardinality. While k-means with nine clusters showed some dice for LGN, increasing k-means to nine clusters did not improve overall performance; in fact, k-means (nc = 9) performed worse than k-means (nc = 7). In contrast, spectral clustering consistently demonstrated superior LGN delineation across configurations, supporting a fair and informative comparison between the methods.

**25. Page 12, Line 239: What statistical test was used? (Not explained in the Materials and Methods.)**

We have addressed this concern by adding a dedicated subsection to the Materials and Methods on statistical analysis. This addition clarifies the statistical frameworks used in the analyses (**Page 13, Line 251**).

**26. Page 12, Line 241: VLP is not mentioned. Did k-means clustering perform significantly better for it or was there no statistically significant difference?**

There was no statistically significant difference in Dice overlap for VLP between k-means and spectral clustering. This was confirmed using the Wilcoxon signed-rank test. We have revised the manuscript to state this explicitly to avoid ambiguity (**Page 18, Line 353**).
**27. Page 13, Line 253: The label maps do not appear to resemble the atlas, particularly in the coronal view (Figure 7b). Please explain.**

The differences between our label maps and the Morel atlas are expected. The Morel atlas is defined by cytoarchitectural boundaries, whereas our parcellation is derived from fiber orientation distribution (FOD) features that capture local white-matter orientation structure and connectivity geometry. As shown in prior study (reference 25), structural connectivity-informed subdivisions do not exhibit a one-to-one correspondence with histological nuclei and may span multiple Morel regions(**Page 12, Line 228**); therefore, exact visual alignment with atlas boundaries is not anticipated. We have added this clarification to the manuscript(**Page 24,Line 467**).  **28. Page 13, Line 256: Does initialization include defining the number of clusters?**

No, it doesn’t need a specific number of clusters. Please refer to Comment 8.

**29. Page 13, Line 257: The four subdivisions are not visible in Fig. S4. It still looks like three.**

We updated Figure S4 (now S8a Fig) by also depicting a coronal slice where all four subdivisions are visible. We refer the reader to the revised Supplementary material for the updated visualization.

**30. Page 13, Line 264: Why were structural connectivity profiles derived in only five subjects (out of 30)? Were they randomly selected?**

Structural connectivity profiles were derived in a randomly selected subset of five subjects. This subsampling was motivated by computational considerations: generating 100 million streamlines per subject is highly resource-intensive and was constrained by available computational resources. We have revised the manuscript to state this explicitly (**Page 12, Line 237**). **31. Page 13, Line 268: Cluster numbers and atlas subnuclei are inconsistent. Should not R3/L2 correspond to PuM, R2/L3 to PuL, and R4/L4 to Pul? Also, Fig. S5 does not show R4 or L4.**

We updated the Figure to match the naming. Please refer to the revised Figure 5 and S9 Fig for the revised naming and visualization.
**32. Page 13, Line 270: In the connectivity matrix (Fig. S5), why is the spectral clustering coupling half that of the atlas?**

For construction of the connectivity matrix, we applied inverse node volume scaling (-scale_invnodevol in MRtrix3’s tck2connectome), which scales each streamline contribution to a connectome edge by the inverse of the volumes of the two connected nodes, thereby reducing size-related biases in streamline counts. As the sizes of our data-driven clusters differ substantially from those in the Morel pulvinar parcellation, differences in the resulting coupling strengths are expected under this scaling. We have added the clarification in the manuscript(**Page 22,Line 417**).

**33. Page 13, Line 271: “strong coupling” is relative. Please be more specific by reporting the actual values. (Applies to the other connections mentioned in this paragraph.)**

We appreciate the reviewer’s suggestion to quantify the term “strong coupling.” In response, we have revised the manuscript (**Page 21, Line 401**) to explicitly report the corresponding connectivity values for all pulvinar–target region associations.

**34. Page 14, Line 278: How exactly is the spectral clustering framework presented here better than Battistella et al., 2017’s (**[**https://doi.org/10.1007/s00429-016-1336-4;**](https://nam11.safelinks.protection.outlook.com/?url=https%3A%2F%2Fdoi.org%2F10.1007%2Fs00429-016-1336-4%3B&data=05%7C02%7Cdebottamad%40arizona.edu%7Cbd6f4ed52fd646cd3e7408de36803743%7C5ee35505eb8e4929937d645df5013288%7C1%7C0%7C639008121481538618%7CUnknown%7CTWFpbGZsb3d8eyJFbXB0eU1hcGkiOnRydWUsIlYiOiIwLjAuMDAwMCIsIlAiOiJXaW4zMiIsIkFOIjoiTWFpbCIsIldUIjoyfQ%3D%3D%7C0%7C%7C%7C&sdata=3KanKHUvOAN51ThsQ4f%2FFYAYf2bn4bfJfhgU4p4opdo%3D&reserved=0) **Reference 8) k-means clustering? What were the specific comparison metrics? Also, were other k-means clustering studies using similar features considered? If so, they should also be discussed. If not, the claim that spectral clustering performs better than k-means clustering is not well supported. It may outperform Battistella et al., 2017’s k-means clustering approach, but not k-means clustering in general.**

We did not intend to claim that spectral clustering is superior to k-means clustering in general. Our goal was to implement and evaluate the k-means–based approach of Battistella et al. (2017) as a baseline, because it is the closest prior work using similar diffusion features (FODs). In the revised manuscript, we therefore frame the contribution as improved robustness and anatomical interpretability relative to Battistella et al. baseline under identical feature inputs, rather than asserting universal superiority over all possible k-means clustering approaches (**Page 19, Line 440**).

**35. Page 14, Line 280: Is the thalamus mask the rate-limiting step or feature extraction? Approximately how long does each process take?**

Thalamus mask is the rate limiting step, with 8 threads Freesurfer takes 3hr15min to process one subject, THOMAS does it in 15 mins.

**36. Page 14, Line 282: The claim that spectral clustering produced more stable parcellations than k-means clustering appears to be mostly based on the qualitative evaluation and therefore is not as convincing as it could be. A formal stability analysis would strengthen this claim. Also, k-means clustering had higher Dice scores in some cases, including for pulvinar, so the claim that spectral clustering consistently produced more anatomically faithful parcellations should be dialed back.**

We agree with the reviewer that qualitative evaluation alone is insufficient to support claims of parcellation stability. We have revised the manuscript to clarify that stability was evaluated using quantitative Dice similarity coefficients, in addition to qualitative inspection, by comparing subject-specific parcellations against multiple reference atlases. This provides a more explicit and reproducible basis for assessing stability beyond visual assessment alone.

We also acknowledge that k-means clustering achieved higher Dice overlap for certain nuclei, including the pulvinar. As Dice scores varied across nuclei and reference atlases, we have revised the manuscript to avoid implying uniformly superior anatomical fidelity of spectral clustering and instead emphasize nucleus- and atlas-dependent differences between the two methods (**Page 23, Lines 488**).

**37. Page 14, Line 294: Random initialization is not necessarily required for k-means clustering in this context. Initialization can be done in a data-driven (Battistella et al., 2017) or atlas-informed manner (Malaga et al., 2023). Regardless of approach, do the number of clusters have to be determined in advance?**

Yes the number of clusters has to be set in advance as mentioned in **Page 9 Line 178** .

**38. Page 15, Line 302: Why not show the unstable parcellations from k-means clustering? This would strengthen the spectral clustering claims.**

We agree that illustrating the less stable k-means outcomes would help contextualize the stability discussion. To address this, we added a dedicated Results subsection on k-means cluster cardinality, where we explicitly report how k-means behavior changes when increasing the number of clusters (e.g., from 7 to 9).

**39. Page 15, Line 303: “retained relative strength” is not a fair description. k-means clustering significantly outperformed spectral clustering for VPL and pulvinar (Figure 6).**

We agree with the reviewer that “retained relative strength” can read as minimizing the cases where k-means performed better. We have therefore revised the manuscript to describe these results more directly. Specifically, we now state that k-means achieved higher Dice overlap for the pulvinar, and we have updated the text accordingly (**Page 20, Line 466**).

With respect to VPL, although the Dice values may appear higher for k-means, our statistical analysis did not indicate a significant difference.

**40. Page 15, Line 311: It would be helpful to see the k-means clustering results alongside the spectral clustering results in Figure 7, like Figures 3-5.**

We have revised the manuscript to show k-means results in Figure 5 (previously Figure 7) along with the Dice in Figure 6.

**41. Page 16, Line 334: Why was the Krauth-Morel atlas chosen for this study? How would the spectral clustering approach fare using other atlases with more or less subnuclei? In other words, could atlas choice affect the findings (Zilberman et al., 2025;** [**https://doi.org/10.1016/j.nicl.2025.103887**](https://nam11.safelinks.protection.outlook.com/?url=https%3A%2F%2Fdoi.org%2F10.1016%2Fj.nicl.2025.103887&data=05%7C02%7Cdebottamad%40arizona.edu%7Cbd6f4ed52fd646cd3e7408de36803743%7C5ee35505eb8e4929937d645df5013288%7C1%7C0%7C639008121481556530%7CUnknown%7CTWFpbGZsb3d8eyJFbXB0eU1hcGkiOnRydWUsIlYiOiIwLjAuMDAwMCIsIlAiOiJXaW4zMiIsIkFOIjoiTWFpbCIsIldUIjoyfQ%3D%3D%7C0%7C%7C%7C&sdata=D1qgg0%2BEtw83jWVMgevYCE%2B46lWeokyaElPXYY2qn%2BM%3D&reserved=0)**)? Also, besides atlas correspondence and histology, subject-specific clinical outcomes could be used to evaluate segmentation results (Malaga et al., 2023).**

Most state-of-the-art methods including Iglesias et al. (Freesurfer), Su et al. (THOMAS), Najdenovska et al. compare themselves with the Morel atlas as it is one of the most well known histological atlas that also incorporates some functional information (calbindin/calmodulin). However, in response to this comment, we expanded the evaluation to include multiple reference atlases rather than relying solely on the Krauth-Morel atlas. Specifically, we now assess the clustering-derived parcellations against the Krauth-Morel, Allen Human Brain, and Saranathan atlases.(**Page 16,Line 314**)

We also note that this study is based on publicly available datasets consisting exclusively of healthy control participants (HCP). As a result, subject-specific clinical variables or outcome measures are not available, and additional validation based on clinical associations is not feasible within this cohort. We acknowledge it as limitation which needs further investigation (**Page 25,Line 499**)
**42. Page 17, Line 360: A table showing which clusters correspond to which subnuclei would help with the anatomical interpretation.**

We concur and have added S1 Table, which summarizes the correspondence between the clustering-derived labels and the atlas-defined subnuclei.

**43. Page 17, Line 361: Given the comments above (no stability analysis performed, only one atlas considered), the statement that “the framework advances beyond centroid-based clustering in stability and biological face validity” comes off as a bit of an overstatement. Adjust the claim accordingly.**

We agree that the original wording was too strong in light of the analyses performed. We have therefore revised the text and the revised wording states more narrowly that, under the evaluation performed in this study, the proposed framework demonstrates improved robustness and anatomical plausibility relative to the modified Battistella et al. k-means baseline, without implying general superiority or conclusions beyond the presented evidence. This revision has been made in the manuscript (**Page 23, Line 488**).
**44. Discussion: Should consider how individual variability may contribute to cluster inconsistency in addition to (limitations of) the clustering approach itself.**

We agree with the reviewer that individual anatomical variability can contribute to cluster inconsistency, independent of the clustering method itself. We have addressed this in the manuscript by noting that our study uses a relatively homogeneous population, where inter-individual variability is expected to be limited compared to aging or clinical cohorts. We further clarify that greater heterogeneity in clinical populations may reflect true anatomical differences rather than methodological instability. We now explicitly state that validation in aging and disease cohorts, together with subject-specific clinical outcomes, will be necessary to disentangle these sources of variability and assess generalizability. These points are now stated explicitly in the manuscript (**Page 25, Line 499**).

**45. Discussion: How do the authors reconcile the fact that k-means clustering performed better (higher Dice score, less variability) for pulvinar specifically, the other structure of interest in this study?**

The higher Dice score for the pulvinar with k-means reflects better overlap with a large, single atlas region rather than a more accurate representation of its internal structure. In our results, both k-means and spectral clustering divided the pulvinar into multiple clusters. When a region is split this way, using only the maximum Dice score tends to favor methods like k-means that produce larger, more contiguous clusters. Spectral clustering instead yielded a finer subdivision of the pulvinar, which is not fully captured by a single-label Dice measure. Therefore, the higher Dice score for k-means should be interpreted as a limitation of the evaluation metric rather than evidence of better pulvinar parcellation. This is vindicated by the significantly better Dice performance in the pulvinar subsegmentation for spectral clustering especially on the Allen Brain Human atlas. These points are now stated explicitly in the manuscript (**Page 23, Line 455**).
**46. Discussion: Given intersubject variability in anatomy, assuming that the atlas is the ground truth is a limitation of this study as it assumes that the atlas’ anatomy is representative of all subjects. This should be discussed further as many different atlases are available (Neudorfer et al., 2024).**

We agree that treating any single atlas as “ground truth” is a limitation in the presence of inter-subject anatomical variability and known differences across atlas definitions. We have revised the Discussion to explicitly acknowledge that any atlas may not be fully representative of all individuals, and that overlap metrics such as Dice should therefore be interpreted as measures of anatomical plausibility and correspondence to a given reference, rather than as definitive measures of absolute accuracy.

To partially address the reliance on a single atlas, we expanded our evaluation beyond the Krauth–Morel atlas to include additional atlases (Allen Human Brain atlas and Saranathan atlas). This comparison suggests that, although absolute overlap values vary across atlases, the broader pattern of cluster–nucleus correspondence is reasonably preserved. At the same time, we avoid claiming comprehensive atlas-independence and emphasize that further validation across a wider range of atlases and cohorts would be needed. These points are now stated in the revised manuscript(**Page 25,Line 485**).
**Minor issues**

All minor comments have been addressed directly in the revised manuscript and are marked as R1CX (Minor), where X denotes the corresponding comment number. Please refer to the manuscript for the specific corrections.

**1. Page 1, Line 10: Different name listed as corresponding author on cover page.**
**2. Page 4, Line 65: Define “MS” in acronym “MSMT-CSD”.**
**3. Page 4, Line 71: Define acronyms “LGN” and “MGN”.**

**4. Page 5, Line 83: Define acronym “DBS”.
5. Page 5, Line 91: Briefly explain what “multi-shell” means.
6. Page 6, Line 103: What field strength was used to acquire the images?
7. Page 7, Line 137: Fig. S1 appears to show the output of the spherical clustering step, not the BIRCH clustering step.
8. Page 8, Line 161: Previous studies are mentioned, but only one study is cited.
9. Evaluation: In-text references to the supplementary figures would be helpful.
10. Page 10, Line 188: Prior studies are mentioned, but only one study is cited
11. Figure 5a: The image resolution is too low to clearly read the values. What do the red boxes indicate?**

**12. Page 11, Line 226: Also report the Dice scores from k-means clustering for easier comparison.
13. Page 12, Line 238: Figure 6 is a bar plot, not a box plot.**

**14. Page 12, Line 249: Do Figure 7a and Fig. S3 show the same five subjects?
15. Fig. S4: Should the colorbar be next to the max probability label maps, like in Figure 7?
16. Page 13, Line 262: Add “mm” after “y = -29”.
17. Page 15, Line 307: Mention some of the limitations of diffusion MRI.
18. Page 15, Line 317: Multimodal work is mentioned, but no reference is provided.
19. Page 17, Line 356: What clinical contexts are being referred to?
20. Page 17, Line 358: What is meant by “a disciplined combination”?
21. Page 17, Line 359: What is meant by “spectral embedding”?
22. Page 20, Line 419: Iglehart et al., 2019’s (**[**https://cds.ismrm.org/protected/19MProceedings/PDFfiles/2707.html;**](https://nam11.safelinks.protection.outlook.com/?url=https%3A%2F%2Fcds.ismrm.org%2Fprotected%2F19MProceedings%2FPDFfiles%2F2707.html%3B&data=05%7C02%7Cdebottamad%40arizona.edu%7Cbd6f4ed52fd646cd3e7408de36803743%7C5ee35505eb8e4929937d645df5013288%7C1%7C0%7C639008121481576871%7CUnknown%7CTWFpbGZsb3d8eyJFbXB0eU1hcGkiOnRydWUsIlYiOiIwLjAuMDAwMCIsIlAiOiJXaW4zMiIsIkFOIjoiTWFpbCIsIldUIjoyfQ%3D%3D%7C0%7C%7C%7C&sdata=svnbRAaQ8InsI6rxjajYhNU3gABZuPeJQzSdpG3GW%2FI%3D&reserved=0) **Reference 12) citation is incomplete.
23. Page 21, Line 468: Left and right (in reference to the columns) are mixed up.
24. Page 21, Line 469: Coronal and axial are mixed up
25. Page 22, Line 479: All slices are axial; none are coronal.
26. Page 22, Line 480: Results from k-means clustering are also shown, not only spectral clustering.
27. Page 22, Line 487: Coronal should be axial.
28. Page 22, Line 492: Panels a and b are listed in the figure caption, but the labels are not on Fig. S5.
29. Figure 1: Is “Crop” only associated with “THOMAS” and “Registered” with “ANTS”? If so, there is a Crop-ANTS combo on the right side of the diagram.**

**30. Figure 6: Add asterisks to indicate statistically significant differences between algorithms.**

**Reviewer #2:**

We thank the reviewer for their constructive comments and have addressed each point below. We have clarified the parameter selection for the spectral clustering framework. In addition, we now explicitly describe the role of initialization, detailing how the supercluster-based initialization stabilizes the spectral embedding and improves consistency across subjects. We have further developed the pulvinar analysis to strengthen the evaluation of our clustering framework. Specifically, we now report Dice overlap results using two independent reference atlases, providing complementary assessments of spatial correspondence. This expanded analysis is presented in the Results section and supported by additional figures, enabling a more comprehensive evaluation of pulvinar subdivision performance. All revisions pertaining to Reviewer 2 have been explicitly labeled as R2CX in the manuscript, where X indicates the comment number; additional editorial comments are tagged as EC.

1. **Parameter selection for spectral clustering requires more justification. The choice of γ = 48 (or 35 for pulvinar) is described as empirical or based on “distance matching”, but the actual selection procedure needs clarification and reproducible detail. How sensitive is the final clustering to small variations in these parameters? Did the authors perform a grid search or sensitivity analysis?**

We had indeed performed a grid search but had omitted the results in the paper for clarity which we have now added as Supplementary data. Briefly, we first examined a range of γ values and evaluated their effect on the final parcellation using maximum probability maps thresholded at 50%. As shown in S2 Fig.(thalamus) and S6 Fig.(pulvinar), the spatial patterns remain stable over γ = 32–64 for the thalamus and γ = 16–64 for the pulvinar. Based on this initial screening, we fixed these ranges as stable operating intervals.

Within these stable ranges, γ was then selected by choosing the value at which the Euclidean distances of the spatial coordinates and the FOD features were closest. This resulted in γ = 48 for the thalamus and γ = 35 for the pulvinar. These results indicate that clustering is not sensitive to small variations in γ within the chosen ranges.

2. **The number of clusters (7 for k-means, 9 for spectral clustering) appears chosen primarily to align with known thalamic nuclei and account for LGN/MGN. Why 9 clusters is appropriate for the MSMT-CSD + SC framework but [not] for the K-means approach?**

Spectral clustering was performed with nine clusters to explicitly account for the LGN and MGN, which are included in the thalamus mask and are known to be separable based on MSMT-CSD–derived features. To be consistent with Batistella’s original work which used 7 clusters and a thalamic mask that did not have LGN/MGN, k-means was evaluated at 7 clusters and 9 clusters and compared to still establish 7 as the optimal number. As discussed in the results section in detail, k-means exhibits a significant reduction in ARI when increasing the number of clusters from seven to nine (p < 0.001), indicating sensitivity to cluster number.

This sensitivity is also reflected in the maximum probability maps thresholded at 50% (S4 Fig), where k-means at nine clusters shows missing voxel labels (gaps), which implies inter-subject instability.

3. **Using BIRCH as a pre-clustering step is novel, but the rationale should be strengthened. Did the authors compare BIRCH + SC vs SC alone?**

We evaluated spectral clustering both with and without the BIRCH pre-clustering step. As shown in S1 Fig, spectral clustering without BIRCH could only be performed stably up to eight clusters. Beyond this, the resulting parcellations looked random.

The intersubject instabilities for SC without initialization are reflected in spatial probabilistic label maps, the ventro-lateral region shows increased fuzziness that indicates boundary overlap across subjects. In contrast, incorporating BIRCH as a pre-clustering step produces more compact nuclei and sharper probabilistic boundaries, indicating improved stability and reproducibility. We have made modifications in the Supplementary document to enhance the clarity.

The advantage of initialization was further observed in the pulvinar parcellation. Without initialization, spectral clustering consistently produced three pulvinar clusters, with the anterior (PuA) and part of medial (PuM) subdivisions merging into a single cluster as shown in S8a Fig. In contrast, when combined with BIRCH initialization, spectral clustering reliably resolved four distinct pulvinar clusters, demonstrating improved separation of pulvinar subregions and highlighting the role of initialization in capturing finer-grained pulvinar organization.

4. **Pulvinar evaluation is interesting but underdeveloped. Four clusters are chosen, but there is no rationale provided relating the number to known subregions in the methods section. Connectivity analysis is described in results but just briefly mentioned in the methods. Overall, pulvinar analysis is just briefly mentioned in the discussion section and recent articles are not reported (10.1002/hbm.22781;** [**https://doi.org/10.7554/eLife.100937.3;**](https://nam11.safelinks.protection.outlook.com/?url=https%3A%2F%2Fdoi.org%2F10.7554%2FeLife.100937.3%3B&data=05%7C02%7Cdebottamad%40arizona.edu%7Cbd6f4ed52fd646cd3e7408de36803743%7C5ee35505eb8e4929937d645df5013288%7C1%7C0%7C639008121481602688%7CUnknown%7CTWFpbGZsb3d8eyJFbXB0eU1hcGkiOnRydWUsIlYiOiIwLjAuMDAwMCIsIlAiOiJXaW4zMiIsIkFOIjoiTWFpbCIsIldUIjoyfQ%3D%3D%7C0%7C%7C%7C&sdata=zuZlebbzpPc1RAFsSsALu105qcoN%2B7acUrPwezSsxdo%3D&reserved=0)[**https://doi.org/10.1002/hbm.70062;**](https://nam11.safelinks.protection.outlook.com/?url=https%3A%2F%2Fdoi.org%2F10.1002%2Fhbm.70062%3B&data=05%7C02%7Cdebottamad%40arizona.edu%7Cbd6f4ed52fd646cd3e7408de36803743%7C5ee35505eb8e4929937d645df5013288%7C1%7C0%7C639008121481624486%7CUnknown%7CTWFpbGZsb3d8eyJFbXB0eU1hcGkiOnRydWUsIlYiOiIwLjAuMDAwMCIsIlAiOiJXaW4zMiIsIkFOIjoiTWFpbCIsIldUIjoyfQ%3D%3D%7C0%7C%7C%7C&sdata=XwBHSfsGU3WNX%2BU0xVmTdTadxJP1Cy2SWcBvCv9h3fI%3D&reserved=0) [**https://doi.org/10.1523/JNEUROSCI.1575-14.2015**](https://nam11.safelinks.protection.outlook.com/?url=https%3A%2F%2Fdoi.org%2F10.1523%2FJNEUROSCI.1575-14.2015&data=05%7C02%7Cdebottamad%40arizona.edu%7Cbd6f4ed52fd646cd3e7408de36803743%7C5ee35505eb8e4929937d645df5013288%7C1%7C0%7C639008121481642908%7CUnknown%7CTWFpbGZsb3d8eyJFbXB0eU1hcGkiOnRydWUsIlYiOiIwLjAuMDAwMCIsIlAiOiJXaW4zMiIsIkFOIjoiTWFpbCIsIldUIjoyfQ%3D%3D%7C0%7C%7C%7C&sdata=QKhn0xLwW3z8qSz%2BcuBNB044TU0DrPqIuMVAA%2BOZXXY%3D&reserved=0)**)
   -How do connection strengths are defined in Fig S5?**

We thank the reviewer for this important feedback. We have now revised the manuscript to strengthen the pulvinar analysis and improve clarity. The rationale for selecting four pulvinar clusters is now explicitly described in the Materials and Methods, adding references to both the Morel atlas and recent connectivity-informed studies which strongly show 4 divisions. The tractography-based connectivity analysis, previously summarized briefly, has also been expanded in the Methods to clearly describe the workflow.

The quantitative and qualitative evaluation of pulvinar clustering is now presented in greater detail in the Results, with the corresponding outcomes shown in Figure 5 and 6, and additional analyses provided in the Supplementary Materials. The discussion has been updated to better contextualize our findings in light of recent pulvinar literature, including the cited studies.

Regarding Figure S5 (current Figure 8), connection strength is defined as the normalized number of streamlines connecting each pulvinar cluster to the relevant cortical target regions. These values were computed using MRtrix tck2connectome with inverse volume weighting, derived at the subject level and then averaged across subjects. This definition has been clarified in the Methods section and in the Supplementary figure caption to improve transparency and reproducibility.

5. **“The findings of this study indicate that the modified spectral clustering method can be effectively incorporated into the THOMAS pipeline to provide a ready-to-use solution for generating four anatomically consistent pulvinar subdivisions in addition to whole thalamus”. It’s not clear how the Authors think to integrate SC into THOMAS.**

This was not exactly what we intended to convey. We have revised the manuscript to clarify that spectral clustering can not be integrated into the THOMAS pipeline per se, but could be applied as an add-on step for pulvinar sub-segmentation. In essence, we can use the accurate whole pulvinar nucleus label from THOMAS as a mask to the SC algorithm to subdivide it into 4 subdivisions.
