# Supplementary material for: Robust thalamic nuclei segmentation using spectral clustering of fiber orientation distributions
Source: PLoS One. 2026 Mar 25;21(3):e0345649. doi: 10.1371/journal.pone.0345649 (PMC13016311; doi:10.1371/journal.pone.0345649)
Supplement: Peer review history

# Peer review: Decision Letter 0

**DOI:** 10.1371/journal.pone.0345649.r001

**Authors:** Signe Bray

8 Dec 2025

Dear Dr. Bilgin,

plosone@plos.org . When you're ready to submit your revision, log on to https://www.editorialmanager.com/pone/ and select the 'Submissions Needing Revision' folder to locate your manuscript file.

- A rebuttal letter that responds to each point raised by the academic editor and reviewer(s). You should upload this letter as a separate file labeled 'Response to Reviewers'.
- A marked-up copy of your manuscript that highlights changes made to the original version. You should upload this as a separate file labeled 'Revised Manuscript with Track Changes'.
- An unmarked version of your revised paper without tracked changes. You should upload this as a separate file labeled 'Manuscript'.

If you would like to make changes to your financial disclosure, please include your updated statement in your cover letter. Guidelines for resubmitting your figure files are available below the reviewer comments at the end of this letter.

If applicable, we recommend that you deposit your laboratory protocols in protocols.io to enhance the reproducibility of your results. Protocols.io assigns your protocol its own identifier (DOI) so that it can be cited independently in the future. For instructions see: https://journals.plos.org/plosone/s/submission-guidelines#loc-laboratory-protocols . Additionally, PLOS ONE offers an option for publishing peer-reviewed Lab Protocol articles, which describe protocols hosted on protocols.io. Read more information on sharing protocols at https://plos.org/protocols?utm_medium=editorial-email&utm_source=authorletters&utm_campaign=protocols .

We look forward to receiving your revised manuscript.

Kind regards,

Signe Bray

Academic Editor

PLOS One

Journal Requirements:

Journal requirements:

When submitting your revision, we need you to address these additional requirements.

1. Please ensure that your manuscript meets PLOS ONE's style requirements, including those for file naming. The PLOS ONE style templates can be found at

https://journals.plos.org/plosone/s/file?id=wjVg/PLOSOne_formatting_sample_main_body.pdf and

https://journals.plos.org/plosone/s/file?id=ba62/PLOSOne_formatting_sample_title_authors_affiliations.pdf

2. Please note that PLOS One has specific guidelines on code sharing for submissions in which author-generated code underpins the findings in the manuscript. In these cases, all author-generated code must be made available without restrictions upon publication of the work. Please review our guidelines at https://journals.plos.org/plosone/s/materials-and-software-sharing#loc-sharing-code and ensure that your code is shared in a way that follows best practice and facilitates reproducibility and reuse.

3. Thank you for stating the following in the Acknowledgments Section of your manuscript:

“We gratefully acknowledge support from the National Institutes of Health (Grant R01 EB032674). The content is solely the responsibility of the authors and does not necessarily represent the official views of the National Institutes of Health. The datasets were provided by the WU Minn Consortium of the Human Connectome Project (PIs David Van Essen and Kamil Ugurbil; Award 1U54 MH091657), funded by the 16 NIH Institutes and Centers of the NIH Blueprint for Neuroscience Research, and by the McDonnell Center for Systems Neuroscience at Washington University in St. Louis.”

We note that you have provided additional information within the Acknowledgements Section that is not currently declared in your Funding Statement. Please note that funding information should not appear in the Acknowledgments section or other areas of your manuscript. We will only publish funding information present in the Funding Statement section of the online submission form.

Please remove any funding-related text from the manuscript and let us know how you would like to update your Funding Statement. Currently, your Funding Statement reads as follows:

“AB, MS: This study was supported by the National Institutes of Health (www.nih.gov) under award number R01 EB032674. The funders had no role in study design, data collection and analysis, decision to publish, or preparation of the manuscript.”

Please include your amended statements within your cover letter; we will change the online submission form on your behalf.

4. Please note that your Data Availability Statement is currently a direct link to each database can’t access. If your manuscript is accepted for publication, you will be asked to provide these details on a very short timeline. We therefore suggest that you provide this information now, though we will not hold up the peer review process if you are unable.

5. If the reviewer comments include a recommendation to cite specific previously published works, please review and evaluate these publications to determine whether they are relevant and should be cited. There is no requirement to cite these works unless the editor has indicated otherwise.

Additional Editor Comments:

Thank your for submitting this work for consideration. Two reviewers have provided feedback. While both felt that the work has value and merit, they raised a number of points that require additional clarification, justification and quantification.

[Note: HTML markup is below. Please do not edit.]

Reviewers' comments:

Reviewer's Responses to Questions

**Comments to the Author**

1. Is the manuscript technically sound, and do the data support the conclusions?

Reviewer #1: Partly

Reviewer #2: Yes

**********

2. Has the statistical analysis been performed appropriately and rigorously?

Reviewer #1: No

Reviewer #2: Yes

**********

3. Have the authors made all data underlying the findings in their manuscript fully available?

The PLOS Data policy

Reviewer #1: Yes

Reviewer #2: No

**********

4. Is the manuscript presented in an intelligible fashion and written in standard English?

Reviewer #1: Yes

Reviewer #2: Yes

**********

Reviewer #1: Summary of the research and overall impression

The manuscript presents a modified clustering framework designed to segment the thalamus into its constituent subnuclei using structural MRI and diffusion-weighted MRI data. Central to the approach is the use of spectral clustering rather than more conventional methods such as k-means, with voxel position and fiber orientation distribution-based features serving as inputs to the algorithm. In addition to whole-thalamus parcellation, the authors extend their method to the segmentation of the pulvinar nucleus, illustrating its potential to delineate smaller and more challenging substructures. The manuscript asserts that this spectral clustering approach offers improved stability and greater anatomical consistency across subjects compared to traditional techniques, and it provides both quantitative (Dice similarity scores and connection strength metrics) and qualitative evidence (maximum probability and spatial probabilistic label maps) in support of these claims. However, the qualitative results outweigh the quantitative analyses, and the Materials and Methods notably lacks a statistical analysis section, leaving the claims insufficiently substantiated. This shortage of quantitative evidence is the manuscript’s most significant limitation. Although spectral clustering is a promising and appropriate method for this application, its advantages over k-means clustering are not convincingly demonstrated, which is problematic given that this is the central argument of the work. For these reasons, I cannot recommend the manuscript for publication in its current form. A major revision is warranted by strengthening the evaluation with more rigorous statistical analyses that clearly establish the improved performance of spectral clustering and/or by tempering the claims regarding its advantages.

Evidence and examples

Major issues

1. Page 2, Line 20: How much does the approach improve stability and anatomical consistency by?

2. Page 2, Line 23: Why does smoothness matter? How was it determined that the parcellations were “more biologically meaningful”?

3. Page 2, Line 24: How many subnuclei in total were evaluated?

4. Page 3, Line 54: What about anatomical variability across atlases (Neudorfer et al., 2024; https://doi.org/10.1016/j.neurot.2023.e00313)?

5. Materials and Methods: Why is there no statistical analysis section?

6. Page 6, Line 101: How many subjects in total are in the HCP Young Adult database? If more than 30, why were only 30 subjects included in this study?

7. Page 7, Line 133: Because it “forms the primary methodological contribution”, what exactly was modified in the spectral clustering framework? The initialization (via BIRCH clustering), the features used (FOD-based), the thalamus mask generation?

8. Page 7, Line 136: How many superclusters are generated? Is that determined in advance?

9. Page 7, Line 141: Seeding does not have to be random for k-means clustering in this context. For example, it can be informed by an atlas (Malaga et al., 2023; https://doi.org/10.1016/j.neurom.2022.09.013).

10. Page 8, Line 159: For the k-means clustering comparison, were the same features used?

11. Page 8, Line 160: Which subnuclei did the seven clusters represent? Based on which atlas?

12. Page 8, Line 161: Why not use nine clusters for k-means clustering? Is it a fair comparison if spectral clustering used nine clusters, while k-means clustering used seven?

13. Page 9, Line 167: Why specifically use four clusters? Which anatomical regions do they represent?

14. Page 9, Line 174: How many subnuclei are in this atlas? (14 based on Figure 4?) Which subnuclei?

15. Page 9, Line 175: What does “visually assess cluster consistency” mean? What exactly was being looked at? How was it determined if the clustering looked reasonable? Consistency is not the same as (anatomical) accuracy.

16. Page 9, Line 183: Why was no similar quantitative analysis using Dice scores performed for pulvinar clustering?

17. Page 10, Line 188: Guedj and Vuilleumier, 2020 (https://doi.org/10.1016/j.neuroimage.2020.117162; Reference 26) found five pulvinar clusters, so why use four in this study?

18. Page 10, Line 200: For k-means clustering, were the seeds randomly initialized, as opposed to being atlas-informed?

19. Page 10, Line 205: Figure 4 highlights the consistency of spectral clustering across subjects; however, k-means clustering also appears consistent. This should be discussed as spectral clustering is not clearly better than k-means clustering based on this specific result.

20. Page 10, Line 207: Similar to Comment 19, consistent parcellations do not appear to be unique to spectral clustering based on Fig. S3.

21. Figure 3: Why not show segmentation results for seven clusters using spectral clustering as well?

22. Figure 5b: What are the subnucleus-number pairs? For example, is VA equivalent to 1? Also, why are 10 subnuclei from the atlas evaluated against seven clusters for k-means clustering and nine clusters for spectral clustering? Is this a fair comparison?

23. Page 11, Line 225: Did spectral clustering produce significantly higher Dice scores for all key thalamic nuclei? This sentence as written suggests so. However, there were instances where k-means clustering performed better.

24. Page 11, Line 228: Given that k-means clustering was limited to seven clusters, was it possible for it to partially recover LGN? Again, is this a fair comparison against spectral clustering? (Partially addressed on Page 12, Line 242.)

25. Page 12, Line 239: What statistical test was used? (Not explained in the Materials and Methods.)

26. Page 12, Line 241: VLP is not mentioned. Did k-means clustering perform significantly better for it or was there no statistically significant difference?

27. Page 13, Line 253: The label maps do not appear to resemble the atlas, particularly in the coronal view (Figure 7b). Please explain.

28. Page 13, Line 256: Does initialization include defining the number of clusters?

29. Page 13, Line 257: The four subdivisions are not visible in Fig. S4. It still looks like three.

30. Page 13, Line 264: Why were structural connectivity profiles derived in only five subjects (out of 30)? Were they randomly selected?

31. Page 13, Line 268: Cluster numbers and atlas subnuclei are inconsistent. Should not R3/L2 correspond to PuM, R2/L3 to PuL, and R4/L4 to Pul? Also, Fig. S5 does not show R4 or L4.

32. Page 13, Line 270: In the connectivity matrix (Fig. S5), why is the spectral clustering coupling half that of the atlas?

33. Page 13, Line 271: “strong coupling” is relative. Please be more specific by reporting the actual values. (Applies to the other connections mentioned in this paragraph.)

34. Page 14, Line 278: How exactly is the spectral clustering framework presented here better than Battistella et al., 2017’s (https://doi.org/10.1007/s00429-016-1336-4; Reference 8) k-means clustering? What were the specific comparison metrics? Also, were other k-means clustering studies using similar features considered? If so, they should also be discussed. If not, the claim that spectral clustering performs better than k-means clustering is not well supported. It may outperform Battistella et al., 2017’s k-means clustering approach, but not k-means clustering in general.

35. Page 14, Line 280: Is the thalamus mask the rate-limiting step or feature extraction? Approximately how long does each process take?

36. Page 14, Line 282: The claim that spectral clustering produced more stable parcellations than k-means clustering appears to be mostly based on the qualitative evaluation, and therefore is not as convincing as it could be. A formal stability analysis would strengthen this claim. Also, k-means clustering had higher Dice scores in some cases, including for pulvinar, so the claim that spectral clustering consistently produced more anatomically faithful parcellations should be dialed back.

37. Page 14, Line 294: Random initialization is not necessarily required for k-means clustering in this context. Initialization can be done in a data-driven (Battistella et al., 2017) or atlas-informed manner (Malaga et al., 2023). Regardless of approach, do the number of clusters have to be determined in advance?

38. Page 15, Line 302: Why not show the unstable parcellations from k-means clustering? This would strengthen the spectral clustering claims.

39. Page 15, Line 303: “retained relative strength” is not a fair description. k-means clustering significantly outperformed spectral clustering for VPL and pulvinar (Figure 6).

40. Page 15, Line 311: It would be helpful to see the k-means clustering results alongside the spectral clustering results in Figure 7, similar to Figures 3-5.

41. Page 16, Line 334: Why was the Krauth-Morel atlas chosen for this study? How would the spectral clustering approach fare using other atlases with more or less subnuclei? In other words, could atlas choice affect the findings (Zilberman et al., 2025; https://doi.org/10.1016/j.nicl.2025.103887)? Also, besides atlas correspondence and histology, subject-specific clinical outcomes could be used to evaluate segmentation results (Malaga et al., 2023).

42. Page 17, Line 360: A table showing which clusters correspond to which subnuclei would help with the anatomical interpretation.

43. Page 17, Line 361: Given the comments above (no stability analysis performed, only one atlas considered), the statement that “the framework advances beyond centroid-based clustering in stability and biological face validity” comes off as a bit of an overstatement. Adjust the claim accordingly.

44. Discussion: Should consider how individual variability may contribute to cluster inconsistency in addition to (limitations of) the clustering approach itself.

45. Discussion: How do the authors reconcile the fact that k-means clustering performed better (higher Dice score, less variability) for pulvinar specifically, the other structure of interest in this study?

46. Discussion: Given intersubject variability in anatomy, assuming that the atlas is the ground truth is a limitation of this study as it assumes that the atlas’ anatomy is representative of all subjects. This should be discussed further as many different atlases are available (Neudorfer et al., 2024).

Minor issues

1. Page 1, Line 10: Different name listed as corresponding author on cover page.

2. Page 4, Line 65: Define “MS” in acronym “MSMT-CSD”.

3. Page 4, Line 71: Define acronyms “LGN” and “MGN”.

4. Page 5, Line 83: Define acronym “DBS”.

5. Page 5, Line 91: Briefly explain what “multi-shell” means.

6. Page 6, Line 103: What field strength was used to acquire the images?

7. Page 7, Line 137: Fig. S1 appears to show the output of the spherical clustering step, not the BIRCH clustering step.

8. Page 8, Line 161: Previous studies are mentioned, but only one study is cited.

9. Evaluation: In-text references to the supplementary figures would be helpful.

10. Page 10, Line 188: Prior studies are mentioned, but only one study is cited

11. Figure 5a: The image resolution is too low to clearly read the values. What do the red boxes indicate?

12. Page 11, Line 226: Also report the Dice scores from k-means clustering for easier comparison.

13. Page 12, Line 238: Figure 6 is a bar plot, not a box plot.

14. Page 12, Line 249: Do Figure 7a and Fig. S3 show the same five subjects?

15. Fig. S4: Should the colorbar be next to the max probability label maps, like in Figure 7?

16. Page 13, Line 262: Add “mm” after “y = -29”.

17. Page 15, Line 307: Mention some of the limitations of diffusion MRI.

18. Page 15, Line 317: Multimodal work is mentioned, but no reference is provided.

19. Page 17, Line 356: What clinical contexts are being referred to?

20. Page 17, Line 358: What is meant by “a disciplined combination”?

21. Page 17, Line 359: What is meant by “spectral embedding”?

22. Page 20, Line 419: Iglehart et al., 2019’s (https://cds.ismrm.org/protected/19MProceedings/PDFfiles/2707.html; Reference 12) citation is incomplete.

23. Page 21, Line 468: Left and right (in reference to the columns) are mixed up.

24. Page 21, Line 469: Coronal and axial are mixed up.

25. Page 22, Line 479: All slices are axial; none are coronal.

26. Page 22, Line 480: Results from k-means clustering are also shown, not only spectral clustering.

27. Page 22, Line 487: Coronal should be axial.

28. Page 22, Line 492: Panels a and b are listed in the figure caption, but the labels are not on Fig. S5.

29. Figure 1: Is “Crop” only associated with “THOMAS” and “Registered” with “ANTS”? If so, there is a Crop-ANTS combo on the right side of the diagram.

30. Figure 6: Add asterisks to indicate statistically significant differences between algorithms.

Reviewer #2: This manuscript presents a diffusion-based thalamic segmentation framework integrating fast structural masking MSMT-CSD for FOD estimation and a modified spectral clustering procedure initialized with BIRCH superclusters. The application to both the whole thalamus and the pulvinar nucleus is timely and relevant, given the limitations of current segmentation approaches and the increasing interest in thalamic nuclei for neuromodulation and connectomic studies.

The work is technically solid and addresses important limitations of previous methods (e.g., QBI ODF noise, limited nuclei in FreeSurfer, sensitivity of k-means). However, several aspects require clarification, additional justification, and improvements in presentation.

-Parameter selection rfor spectral clustering requires more justification. The choice of γ = 48 (or 35 for pulvinar) is described as empirical or based on “distance matching”, but the actual selection procedure needs clarification and reproducible detail. How sensitive is the final clustering to small variations in these parameters? Did the authors perform a grid search or sensitivity analysis?

-The number of clusters (7 for k-means, 9 for spectral clustering) appears chosen primarily to align with known thalamic nuclei and account for LGN/MGN. Why 9 clusters is appropriate for the MSMT-CSD + SC framework but for the K-means approach?

-Using BIRCH as a pre-clustering step is novel, but the rationale should be strengthened. Did the authors compare BIRCH + SC vs SC alone?

-Pulvinar evaluation is interesting but underdeveloped. Four clusters are chosen, but there is no rationale provided relating the number to known subregions in the methods section. Connectivity analysis is described in results but just briefly mentioned in the methods. Overall, pulvinar analysis is just briefly mentioned in the discussion section and recent articles are not reported (10.1002/hbm.22781; https://doi.org/10.7554/eLife.100937.3; https://doi.org/10.1002/hbm.70062; https://doi.org/10.1523/JNEUROSCI.1575-14.2015)

-How do connection strengths are defined in Fig S5?

-“The findings of this study indicate that the modified spectral clustering method can be effectively incorporated into the THOMAS pipeline to provide a ready-to-use solution for generating four anatomically consistent pulvinar subdivisions in addition to whole thalamus”. It’s not clear how the Authors think to integrate SC into THOMAS.

**********

what does this mean? ). If published, this will include your full peer review and any attached files.

If you choose “no”, your identity will remain anonymous but your review may still be made public.

**Do you want your identity to be public for this peer review?** For information about this choice, including consent withdrawal, please see our Privacy Policy

Reviewer #1: **Yes:** Karlo A. Malaga

Reviewer #2: No

**********

[NOTE: If reviewer comments were submitted as an attachment file, they will be attached to this email and accessible via the submission site. Please log into your account, locate the manuscript record, and check for the action link "View Attachments". If this link does not appear, there are no attachment files.]

To ensure your figures meet our technical requirements, please review our figure guidelines: https://journals.plos.org/plosone/s/figures

You may also use PLOS’s free figure tool, NAAS, to help you prepare publication quality figures: https://journals.plos.org/plosone/s/figures#loc-tools-for-figure-preparation.

NAAS will assess whether your figures meet our technical requirements by comparing each figure against our figure specifications.

# Author comment: Author response to Decision Letter 1

**DOI:** 10.1371/journal.pone.0345649.r002

3 Feb 2026

Our response to the editor and reviewer comments have been uploaded as attachments.

Attachment Submitted filename: Response_to_Reviewers.docx

# Peer review: Decision Letter 1

**DOI:** 10.1371/journal.pone.0345649.r003

**Authors:** Signe Bray

10 Mar 2026

Robust thalamic nuclei segmentation using spectral clustering of fiber orientation distributions

PONE-D-25-55495R1

Dear Dr. Saranathan,

We’re pleased to inform you that your manuscript has been judged scientifically suitable for publication and will be formally accepted for publication once it meets all outstanding technical requirements.

Within one week, you’ll receive an e-mail detailing the required amendments. When these have been addressed, you’ll receive a formal acceptance letter and your manuscript will be scheduled for publication.

An invoice will be generated when your article is formally accepted. Please note, if your institution has a publishing partnership with PLOS and your article meets the relevant criteria, all or part of your publication costs will be covered. Please make sure your user information is up-to-date by logging into Editorial Manager at Editorial Manager®  and clicking the ‘Update My Information' link at the top of the page. For questions related to billing, please contact billing support .

If your institution or institutions have a press office, please notify them about your upcoming paper to help maximize its impact. If they’ll be preparing press materials, please inform our press team as soon as possible -- no later than 48 hours after receiving the formal acceptance. Your manuscript will remain under strict press embargo until 2 pm Eastern Time on the date of publication. For more information, please contact onepress@plos.org.

Kind regards,

Signe Bray

Academic Editor

PLOS One

Additional Editor Comments (optional):

Reviewers' comments:

Reviewer's Responses to Questions

**Comments to the Author**

Reviewer #1: All comments have been addressed

Reviewer #2: All comments have been addressed

**********

2. Is the manuscript technically sound, and do the data support the conclusions?

Reviewer #1: Yes

Reviewer #2: Yes

**********

3. Has the statistical analysis been performed appropriately and rigorously?

Reviewer #1: Yes

Reviewer #2: Yes

**********

4. Have the authors made all data underlying the findings in their manuscript fully available?

The PLOS Data policy

Reviewer #1: Yes

Reviewer #2: No

**********

5. Is the manuscript presented in an intelligible fashion and written in standard English?

Reviewer #1: Yes

Reviewer #2: Yes

**********

Reviewer #1: (No Response)

Reviewer #2: The authors have adequately addressed the concerns raised in the previous review. The revised manuscript now provides clearer justification for the parameter selection in the spectral clustering framework, including the addition of sensitivity analyses and supplementary figures demonstrating the robustness of the clustering results. The rationale for the choice of cluster numbers in both the spectral clustering and k-means approaches has been clarified, and the discussion regarding the inclusion of LGN and MGN is now more transparent.

The explanation of the BIRCH pre-clustering initialization and its impact on the stability of spectral clustering has also been strengthened, with additional supporting analyses provided in the supplementary material. Furthermore, the pulvinar analysis has been expanded and better contextualized within the existing literature, with clearer methodological descriptions and improved evaluation using independent atlases.

Overall, the revisions have significantly improved the clarity and completeness of the manuscript, and the authors have made a good effort to address the reviewer’s comments.

**********

what does this mean? ). If published, this will include your full peer review and any attached files.

If you choose “no”, your identity will remain anonymous but your review may still be made public.

**Do you want your identity to be public for this peer review?** For information about this choice, including consent withdrawal, please see our Privacy Policy

Reviewer #1: **Yes:** Karlo A. Malaga

Reviewer #2: No

**********

# Editor report: Acceptance letter

**DOI:** 10.1371/journal.pone.0345649.r004

**Authors:** Signe Bray

PONE-D-25-55495R1

PLOS One

Dear Dr. Saranathan,

I'm pleased to inform you that your manuscript has been deemed suitable for publication in PLOS One. Congratulations! Your manuscript is now being handed over to our production team.

At this stage, our production department will prepare your paper for publication. This includes ensuring the following:

* All references, tables, and figures are properly cited

* All relevant supporting information is included in the manuscript submission,

* There are no issues that prevent the paper from being properly typeset

You will receive further instructions from the production team, including instructions on how to review your proof when it is ready. Please keep in mind that we are working through a large volume of accepted articles, so please give us a few days to review your paper and let you know the next and final steps.

Lastly, if your institution or institutions have a press office, please let them know about your upcoming paper now to help maximize its impact. If they'll be preparing press materials, please inform our press team within the next 48 hours. Your manuscript will remain under strict press embargo until 2 pm Eastern Time on the date of publication. For more information, please contact onepress@plos.org.

You will receive an invoice from PLOS for your publication fee after your manuscript has reached the completed accept phase. If you receive an email requesting payment before acceptance or for any other service, this may be a phishing scheme. Learn how to identify phishing emails and protect your accounts at https://explore.plos.org/phishing.

If we can help with anything else, please email us at customercare@plos.org.

Thank you for submitting your work to PLOS ONE and supporting open access.

Kind regards,

PLOS ONE Editorial Office Staff

on behalf of

Dr. Signe Bray

Academic Editor

PLOS One
